# Characterization of percutaneous absorption of calcium, magnesium, and potentially toxic elements in two tailored sulfurous therapeutic peloids: a comprehensive in vitro pilot study

**DOI:** 10.1007/s00484-024-02644-2

**Published:** 2024-03-01

**Authors:** Carla Marina Bastos, Fernando Rocha, Carla Patinha, Paula Marinho-Reis

**Affiliations:** 1 Department of Geosciences, GeoBioTec Research Centre, University of Aveiro, Aveiro, 3810-193 Portugal; 2 Institute of Earth Sciences (ICT), Pole of the University of Minho, University of Minho, Braga, 4710-057 Portugal; 3 Exatronic, Aveiro, Lda. 3800-373 Portugal

**Keywords:** Pelotherapy, Clays, Peloid, Transdermal delivery, Franz diffusion

## Abstract

Pelotherapy treatments in thermal spas, which utilize peloids composed of clay minerals mixed with saltwater or mineral-medicinal water, can have various effects on spa users, ranging from therapeutic to potential adverse reactions. Despite the widespread use of peloids, comprehensive information on the penetration and permeation of essential and potentially toxic elements into deeper layers of the skin during pelotherapy is limited. Understanding the concentrations of these elements is crucial for evaluating therapeutic benefits and ensuring safety. This study investigates the in vitro availability and absorption of calcium, magnesium, and potentially toxic elements in two peloids, considering their formulation matrix. To replicate the pelotherapy methodology, an in vitro permeation experiment was conducted using a vertical diffusion chamber (Franz cells) and a biological system with human skin membranes from five Caucasian women, age range between 25 and 51 years. The experiment involved heating the peloids to 45℃. The results emphasize the possible transport properties of chemical elements in peloids, providing valuable information related to potential therapeutic efficacy and safety considerations. Despite no apparent differences between peloids’ chemical composition, the method identified permeation variations among chemical elements. The methodology employed in this study adheres to the guidelines outlined by OECD for analyzing skin absorption through an in vitro approach. Furthermore, it aligns with the associated OECD guidance document for conducting skin absorption studies. The replicability of this methodology not only facilitates the analysis of peloids pre-formulation but also provides a robust means to evaluate the effectiveness of therapeutic elements during topical administration, particularly those with potential toxicity concerns.

**Supplementary Information:**

The online version contains supplementary material available at 10.1007/s00484-024-02644-2.

## Introduction

In the current regulatory framework for the use of cosmetics, it is of utmost importance to assess and address the safety risks associated with the application of therapeutic muds, also known as peloids. This involves the identification of potential hazards stemming from both biological and mineral elements. In pelotherapy treatments for various medical conditions, users may be exposed to adverse effects resulting from chemical factors, such as the presence of hazardous trace elements, and microbiological factors, including the presence of bacteria. Therefore, it is crucial to conduct studies that investigate the presence and mobility of potentially toxic elements in the clay-water mixture used in pelotherapy treatments. These elements may include substances such as arsenic, lead, mercury, cadmium, selenium, antimony, copper, and zinc, among others (Carretero 2002).

The utilization of peloids in thermal centres and spas has been increasing, and these products exhibit a wide range of variations. They are typically composed of a combination of clay minerals, salt water from the sea or a lake, mineral-medicinal water, and occasionally paraffin (Carretero 2002). However, it is challenging to categorize peloids definitively as either cosmetic or medicinal products, as they can meet the criteria for both categories. Consequently, they are often referred to as “borderline products” due to the unique nature of the regulations governing them. They cannot be classified simultaneously under both categories. Therefore, these products require a more rigorous case-by-case evaluation and must comply with the relevant legislation for medicinal products, particularly when used for therapeutic purposes (Nomicisio et al. 2023; Bastos and Rocha 2022; Tian et al. 2022).

The scientific literature has extensively explored the application of clay minerals in skin drug delivery, primarily due to their notable therapeutic properties. These properties include anti-inflammatory effects, wound healing capabilities, antibacterial properties, and their use in cosmetics and sunscreens (Bastos et al. 2022; Cao et al. 2022; Viseras et al. 2019). Nevertheless, there is currently a dearth of scientific information concerning the quality, regulatory compliance, and efficacy of peloids as a topical drug substance (Bastos and Rocha 2022; Massaro et al. 2018). There is a substantial knowledge gap when it comes to understanding the therapeutic effects of pelotherapy. Limited information is available concerning the physicochemical properties of the topical formulation, the precise types of peloids employed, and the concentration of elements involved. Additionally, the extent to which these elements can penetrate and permeate into deeper layers of the skin is not well-established (Bastos et al. 2023; Metshein et al. 2023; Muñoz et al. 2015).

The peloid can be classified as a topical drug product since it is applied to the skin for local action, primarily interacting with the skin surface. However, considering the pelotherapy protocols, it can also be categorized as a transdermal patch or a transdermal delivery system due to its effect on the stratum corneum (s.c.). Understanding its impact on stratum corneum is crucial in defining the dosage form and predicting the anticipated therapeutic outcome (Bastos et al. 2022). The s.c. of the epidermis plays a crucial role as the primary barrier to the dermal absorption of substances. To investigate the permeation of substances through the skin, different types of skin preparations can be utilized. These include split-thickness, full-thickness, and heat-separated epidermis (HSE), each with its advantages based on the specific objectives of the study (Kovács et al. 2021).

According to the OECD guideline, the amount of a substance that permeates the skin and the amount present in the skin are considered potentially absorbable doses. However, the content of the substance in the stratum corneum (s.c.) is not considered fully systemically available, due to the reservoir effect of substances in the skin and their continued diffusion within the skin. The skin can act as a reservoir for a test substance in different layers if it accumulates in the skin instead of directly entering the bloodstream. This depends on the substance’s properties and its interaction with skin components, such as proteins. After exposure, the test substance may continue to diffuse into the skin and become available systemically. It can enter the bloodstream and potentially have effects throughout the body via the lymphatic or sanguine capillary network in the dermis (OECD 2004, b).

The effectiveness of the therapeutic response, in terms of both duration and magnitude, for any topical formulation is determined through three sequential processes: drug release, penetration/diffusion of the active substance through the s.c. and other skin layers, and finally, the achievement of the therapeutic effect. These processes can yield variable outcomes in terms of the safety and efficacy of the formulation (Shah et al. 2015).

In a study conducted by Sánchez-Espejo et al. (2015), the in vitro release of exchangeable cations (Na, K, Mg and Ca) from two therapeutics muds used in Italian and Tunisian spas was investigated. The mud underwent a three-month maturation process in Spanish mineral-medicinal water before being tested using Franz diffusion cells. In these tests, a dialysis membrane separated the donor and receptor chambers, with a diffusion area of 0.64 cm^2^. The study revealed that the in vitro release of cations was lower than the cation exchange capacity (CEC) of the mud (Sánchez-Espejo et al. 2015). Values ranging from 7 to 28 meq/100 g were observed in the in vitro release of exchangeable cations from several Tunisian mudpacks (Khiari et al. 2019).

As peloids are a semi-solid formulation, comparable to ointments, gels, or creams, the appropriate testing method for their permeation study is the vertical diffusion cell (VDC) system, following the Organization for Economic Co-operation and Development (OECD) guidelines (OECD 2004b). The study design for permeation should follow the general recommendations outlined by the OECD, while also considering the specific characteristics of the product being investigated. It is important to note that since there are well-established and validated methodologies, it is possible to use them specifically for studying the permeation of therapeutic muds, but any modifications made to the OECD recommendations must be thoroughly documented.

This study aims to characterize the percutaneous absorption of calcium (Ca), magnesium (Mg), and potentially toxic elements (PTEs) in two tailored sulfurous therapeutic peloids. Investigating skin absorption of PTEs was deemed crucial to assure the safe use of these materials. Assessing the potential for skin absorption of Mg and Ca aimed to produce new evidence about their potential role in the therapeutic properties of the peloids. The study covers various phases of permeation investigation, including quantification and data analysis. Moreover, it aims to align with the objectives delineated in the OECD (2004a, b) guidelines for the vertical diffusion cell (VDC) system. The methodology used in this study follows the guidelines set out in OECD Test No. 428 for the analysis of skin absorption using an in vitro approach (OECD 2004a). Additionally, it complies with the associated OECD guidance document for conducting skin absorption studies (OECD 2011).

## Materials and methods

### Materials

#### Peloids

Two peloids, resulting from an experimental maturation conducted for 90 days, using Portuguese bentonitic clay (Benvila, Portalegre district) and two different Portuguese mineral-medicinal waters were used. The waters were collected from the watershed that supplies the Cró Hotel & Thermal Spa (Sabugal, Guarda district) and the Caldas da Rainha Thermal Hospital (Caldas da Rainha, Leiria district) (Table S1, at supplementary information). The Caldas da Rainha sulfurous water is characterized by its hypersaline mineralization, with chloride and sodium as predominant ions. The Cró sulfurous water is characterized by weak mineralization, with bicarbonate and sodium as predominant ions. The bentonitic clay used is smectitic and the main exchangeable cation is calcium. The maturation of Benavila bentonite with these two waters led to some chemical and mineralogical changes. The smectite content in peloids CR and CRO almost doubled after 90 days, exceeding the initial content of bentonitic clay. Additionally, the exchangeable cations Ca and Mg decreased, while K and Na increased. The trace element composition of Benavila bentonite is noteworthy, considering the safety limits established for cosmetic usage. However, there is concern regarding nickel, as it exceeds the safety limit of 25 ppm. Notably, Benavila bentonite exhibited no detectable amounts of As, Cd, Cr, Mn, Sb, or V. Furthermore, the concentrations of Pb, Cu, Mo, and Zn were below the limit values of 10 ppm, (Pb), 250 ppm (Mo), and 1300 ppm (Zn). All of these trace elements in Caldas da Rainha and Cró peloids showed alterations in their content, requiring an accurate safety analysis. These peloids exhibited technological properties such as good skin adhesion easy handling, large cation exchange capacity, and a low cooling rate, making them suitable for pelotherapy (Bastos and Rocha 2023).

#### Human skin membrane

The skin samples used in the tests were removed from the abdominal region of five Caucasian women who had undergone cosmetic surgery, specifically abdominoplasty. The age range of the skin donors spanned from 25 to 51 years old (Table S2).

The protocol for collecting and handling biological samples obtained prior approval from the Ethics Committee of the Hospital, located in Porto, Portugal. The laboratory that conducted the tests using the Franz diffusion cells strictly followed the approved protocol. Before acquiring the skin, all donors were presented with a “Free and Informed Consent Document”.

#### Receptor fluid

The selected receptor fluid was a Phosphate-buffered saline (PBS) solution with a pH of 7.4, free of Ca^2+^ and Mg^2+^, designed to mimic physiological conditions and maintain sink conditions. This PBS solution was used to suspend the peloid. The PBS is introduced in the receptor chamber and continuously agitated throughout the assay. Before the placement in the receiver compartment, it was subjected to sonication.

#### Diffusion cell system

The in vitro permeation test was performed using the diffusion cell system Franz SES GMBH, Model V6A-02 manufactured by SES GmbH – Anlytical Systems, Germany. To assess the Transepidermal Water Loss (TEWL), the Aquaflux device (AF200, Biox, United Kingdom), equipped with adapters precisely matching the size of the donor chamber of the Franz cell, was employed.

The materials and equipment used is detailed in the supplementary information in Table S3 and following the general descriptions in United States Pharmacopeia, USP General Information of < 1724 > Semisolid Drug Products – Performance Tests and USP 37-NF32 (USP, 2014).

### Methods

To ensure the reliability and relevance of this study, careful consideration has been given to the recommendations presented in the SCCS notes of guidance for the testing of cosmetic ingredients and their safety evaluation (SCCS 2010), Annex 1 of the Guideline on Quality of Transdermal Patches (EMA 2014), and draft guideline on quality and equivalence of topical products (EMA 2018). These guidelines and recommendations serve as reference points, validating the accuracy and authenticity of the experimental protocols and analyses conducted throughout the study.

#### Vertical diffusion cell

**Fig. 1:**
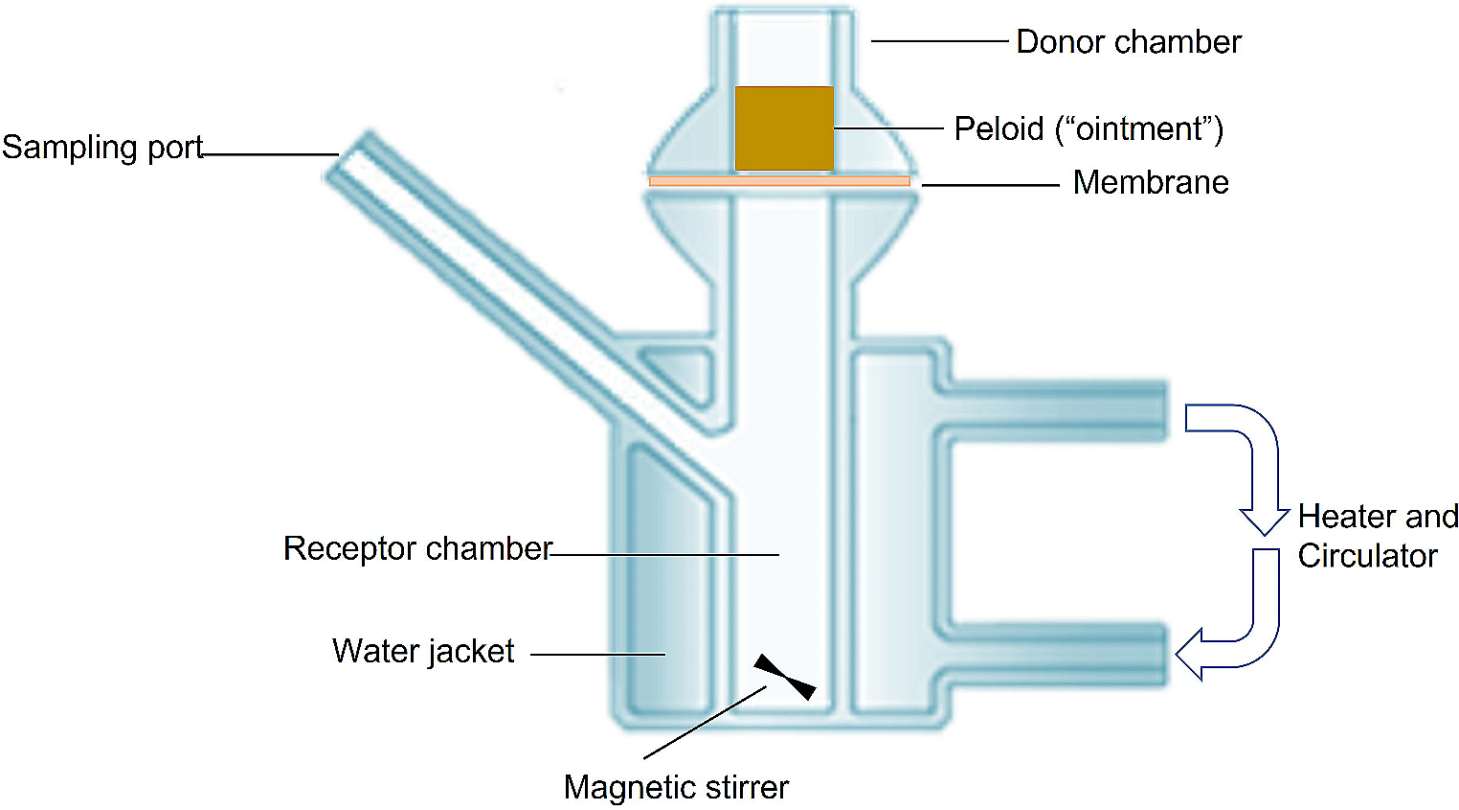
Schematic representation of Franz cells

The skin permeation method selected for this study is based on the utilization of biological membranes within Franz cells at physiological temperature. The experimental setup involved a vertical diffusion chamber (VDC), coupled to a biological system consisting of a human skin membrane. Figure 1 provides a representation of the Franz cells used in the in vitro permeation assays. To conduct the experiment, the peloid was applied onto the surface of an excised skin membrane, serving as a barrier between the two compartments of a diffusion chamber. In the receptor chamber, small samples (aliquots) of the receptor fluid were collected at specific time intervals. These collected aliquots were later analysed to identify and quantify the peloids chemical elements.

The accurate selection of parameters is of utmost importance for precise in vitro skin permeation assays. In this study, the key parameters taken into consideration include the configuration of the Franz cells, the type of membrane employed, the composition of the receptor fluid, the quantity of the sample applied, and the timings for collecting the receptor fluid. These parameters were carefully chosen to ensure the significance and dependability of the results, as well as their applicability to real-life therapeutic and cosmetic contexts.

These methods pertain to in vitro skin permeation assays conducted using Franz diffusion cells with biological membranes maintained at a physiological temperature of 32℃ ± 1, similar to skin temperature. The selection of the finite dose was based on the typical duration of peloid skin contact in pelotherapy, which typically ranges from 20 to 30 min. The peloid was applied uniformly onto the skin surface as a finite dose with an exposure time of 60 min.

In this study, the selected receiving solution was PBS pH 7.4 because it mimics the ionic strength and pH of human plasma and remains relatively stable throughout the experiment. It does not contain surfactants and solubilizers that could potentially interfere with the permeation of the analyte across the membrane. The solubility of the analyte in the receptor fluid was also crucial to ensure that an adequate amount of the peloid was available for diffusion into the receptor chamber for the analysis of cations and trace elements (Carretero and Pozo 2009, 2010; Tateo and Summa 2007).

The results of the chemical analysis of the PBS show that the chemical composition of this receptor fluid aligns with expectations. There are no detectable levels of calcium (Ca) and magnesium (Mg), both falling below their respective detection limits (0.30 mg/L for Ca and 0.10 mg/L for Mg). Phosphorus (P) is the most abundant element in PBS at 425.60 g/L, followed by sodium (Na) at 5.08 g/L.

Quality assurance control of PBS was conducted to detect potential toxic elements, and the following trace elements were found to be below the detection limit (Table S4):

- Silver (Ag), arsenic (As), beryllium (Be), cadmium (Cd) and lithium (Li) (< 2.00 µg/L);
- Aluminium (Al) (< 13.50 µg/L).
- Barium (Ba) (< 5.00 µg/L).
- Cobalt (Co), copper (Cu), nickel (Ni), titanium (Ti), uranium (U) and vanadium (V) (< 1.00 µg/L).
- Iron (Fe), tungsten (W) and zinc (Zn) (< 10.00 µg/L).
- Manganese (Mn) (< 1.50 µg/L).

However, hazardous elements such as chromium (Cr) were detected above the detection limit.

The methodology used in this study required a minimum sample volume of 5 mL corresponding to the total capacity of the Franz cell receiving chamber.

#### Biological membranes

According to the OECD guidelines in vitro skin absorption tests should be conducted using human skin as the biological membrane, which is considered the “gold standard membrane” for this type of testing. These studies are designed to examine the permeation of substances through intact (non-injured) skin, thereby excluding any potential influence of skin lesions that could enhance the permeation of compounds.

Before the experiments, the skin underwent a thorough examination to identify and exclude areas with injuries or any type of lesions, tattoos, stretch marks, or an excessive number of hair follicles.

In this study, the HSE method was considered suitable for the permeation screening objectives as it allows using only the s.c. and epidermis as the biological membrane. Therefore, the skin preparation method employed was heat-separated epidermis (HSE) (OECD 2004b).

After the excision of the tissue, the skin specimens were carefully placed in dry containers and kept frozen at -20 °C to maintain the integrity of the specimens until they could be transported and stored. The time elapsed between the surgery and the preparation of the skin was less than twelve hours. At the laboratory, the tissue was thoroughly cleaned, any excess moisture was carefully absorbed, and the hypodermis was removed by the HSE method. The samples, despite being frozen, adhered to the guidelines’ recommendations and were not kept frozen for more than two months. However, this could be a limitation of this study. It is important to note that in vitro studies using frozen skin preparations may not accurately reflect the biotransformation processes that take place in living skin tissue. This is because these preparations lack active enzyme systems for metabolizing the substance.

Initially, the frozen tissue was thawed at room temperature, followed by immersion in a water bath set at 60 °C ± 2 for 90 s. After a 30-second interval at room temperature, the surface of the skin was dried, and the epidermis was carefully separated from the rest of the tissue using tweezers. During this process, special care was taken to preserve the integrity of the epidermis.

The isolated epidermis was then placed in the diffusion cells, ensuring that the s.c. faced upwards towards the donor compartment. The membranes were kept in place for a minimum of 30 min before the experiment, allowing for the proper equilibrium of the system.

Finally, the Franz cells were assembled, and a Parafilm® seal was used to occlusion the components, assuring a tight and leak-proof connection, avoiding evaporation and changes of the receiving solution.

After the preparation of the membranes, a thorough visual inspection was conducted using a magnifying glass mounted on a lamp. Any membranes displaying signs of rupture or minor alterations were promptly discarded to ensure the reliability of the experiment.

The present study included measurements of Transepidermal Water Loss (TEWL) as an essential parameter reflecting the skin’s barrier function, with lower values indicating better barrier integrity (preferably below 10).

TEWL measurements were taken both prior to the application of the peloid and after the 24 h test period. By comparing these measurements, any potential changes could be detected and quantified. In addition to the objective measuring of TEWL, all membranes underwent a visual inspection.

#### Experimental design

The utilization of therapeutic mud in pelotherapy treatments involves applying mud either through poultices or baths, with a typical duration of 20 and 30 min. The mud is heated to 45 °C, which induces perspiration and dilates blood vessels when in contact with the skin. It is understood that the ionic alterations present in the mud permeate the skin, and the duration of application is justified by these physiological phenomena (Bastos et al. 2023).

Two assays were conducted for Cró peloid (CRO), and three assays were conducted for Caldas da Rainha peloid (CR), with different donor membranes, running in parallel. This resulted in a total of twelve cells per assay. Sample distribution for each assay is presented in Table 1.

**Table 1:** Franz cell distribution by donor membrane and peloid type

	Cell 1	Cell 2	Cell 3	Cell 4	Cell 5	Cell 6
Assay 1 Donor age:	CRO 51 A	CRO 51 A	No peloid 51 A	CRO 51 A	CRO 51 A	CRO 51 A
Assay 2 Donor age:	CRO 40 A	CRO 40 A	CRO 25 A	CRO 25 A	No peloid 40 A	No peloid 25 A
Assay 3 Donor age:	CR 36 A	CR 36 A	CR 36 A	CR 36 A	No peloid 36 A	CR 36 A
Assay 4 Donor age:	Unused cell	CR 32 A	CR 32 A	No peloid 32 A	Unused cell	Unused cell
Assay 5 Donor age:	Unused cell	CR 41 A	CR 41 A	No peloid 41 A	Unused cell	Unused cell

After each assay, quality control procedures were implemented to ensure the cleanliness of the Franz cells and assess the efficiency of the washing process. This step involved performing a one-hour wash of the Franz cells to validate the effectiveness of the washing procedure and eliminate any potential contamination that could influence the subsequent analyses and compromise the accuracy of the results. The cells were mounted on their support and the stirring magnets were activated at 500 rpm to facilitate the washing process.

An appropriate assay duration is crucial to enable the collection of components that penetrate the deep layers of the epidermis into the receiving solution. Accurately simulating real-life situations often requires extended time periods, as surface washing of the skin does not always eliminate components that have already deeply penetrated the tissue. Therefore, in this study, the contact time between the mud and the membrane was established as 60 min to ensure ample permeation and effective collection of peloid components in the receiving solution.

**Fig. 2:**
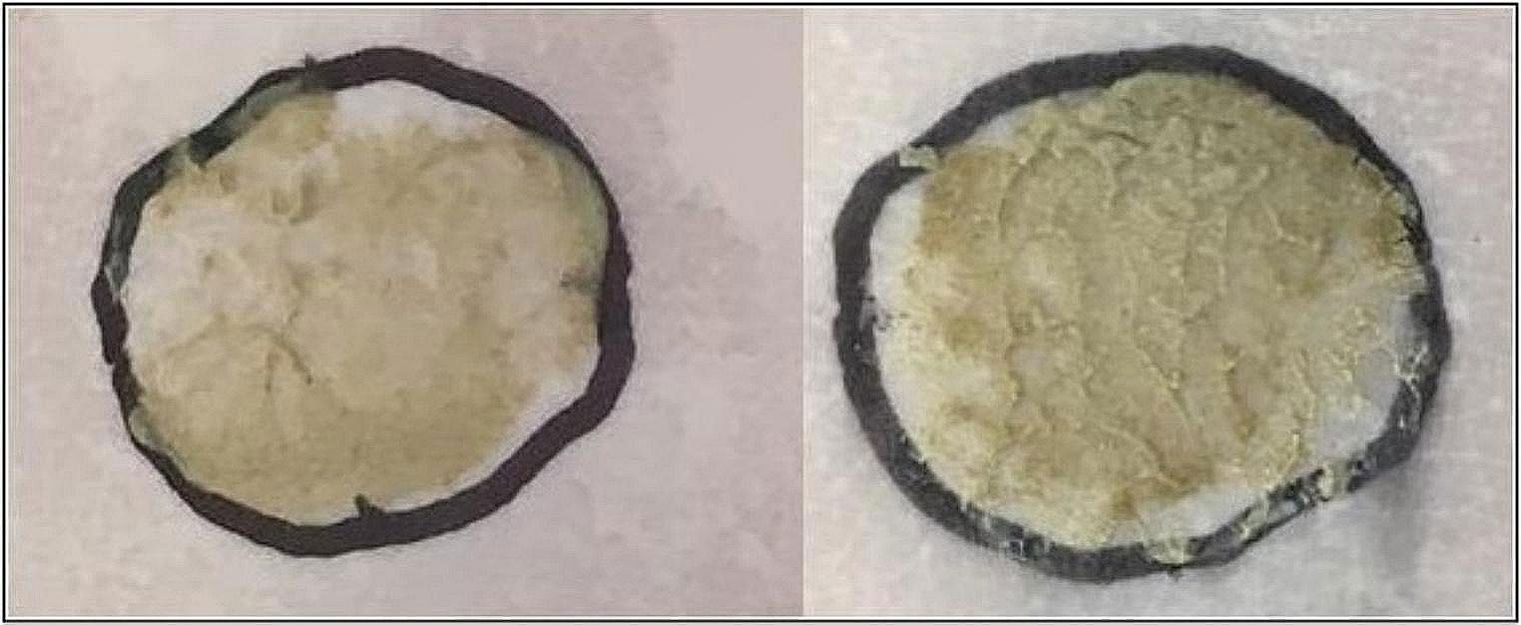
Evaluation of sample quantities on the biological membrane (left: ≅ 17.4 mg; right: ≅ 30 mg)

To ensure uniform coverage of the membrane surface, a precise amount of mud was visually tested for application, as depicted in Fig. 2. The sample amount in the donor chamber ranged from 30 to 60 mg (Table S5).

The peloid was heated in a water bath until a 45℃ temperature before its placement in the donor chamber. To ensure optimal transfer and uniform distribution across the membrane surface, the peloid was carefully placed on top of the plunger of a syringe and then applied. The plunger was weighed before and after the procedure to determine the mass of peloid that remained unused or unapplied. The experimental environmental conditions were between 20 and 25℃.

**Fig. 3:**
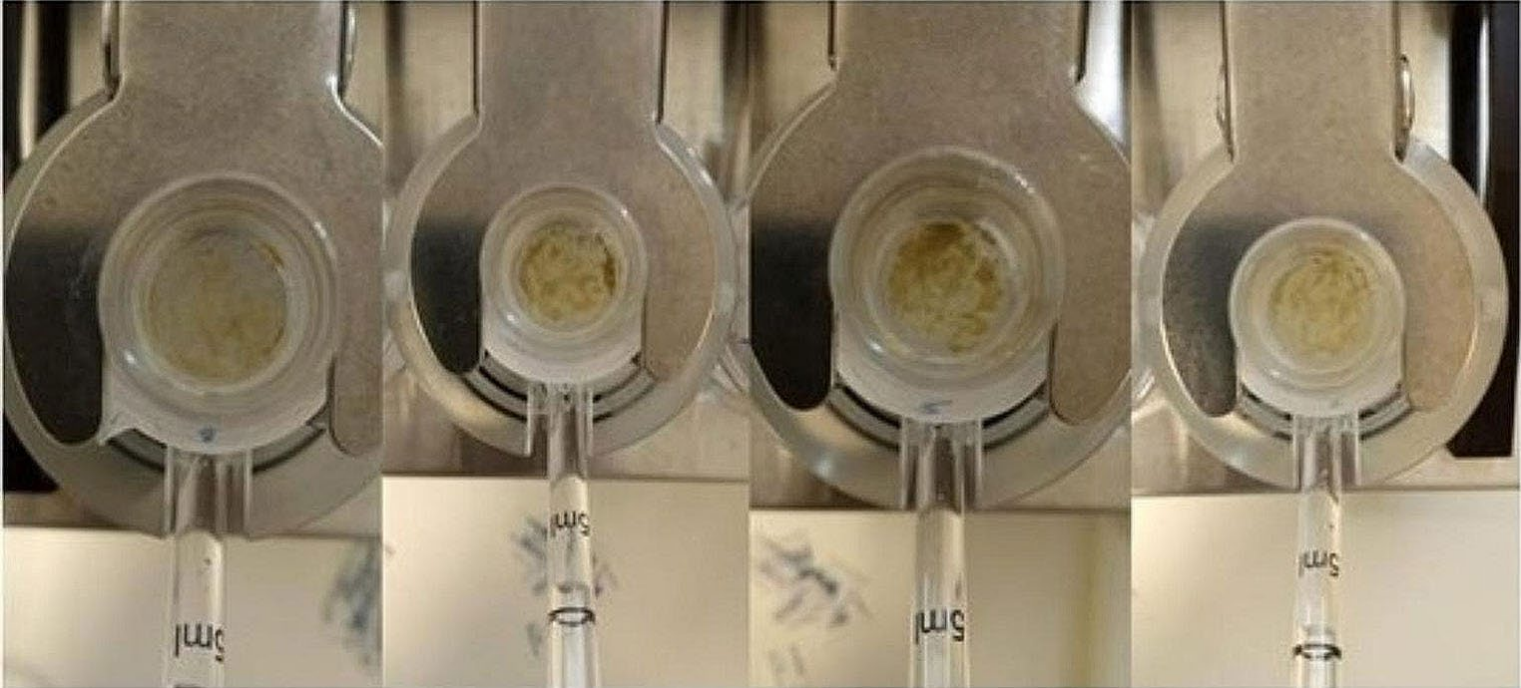
Experimental setup and sample application

Following a one-hour contact between the peloid and the membrane (as shown in Fig. 3), the peloid was removed by washing the biological membrane five times with 1 mL of PBS. To ensure the thorough removal of any remaining peloid residue from the membrane, extensive washing was performed, and the membrane was visually examined.

After the washing, it was observed that a few of the membranes (cell 2 from assay 1 and cell 5 from assay 2) were damaged and no longer intact (Table S6). However, although damaged, cell 5 of assay 2 served as a negative control and could still be included in the subsequent analysis. The system was maintained under the previously established conditions throughout the entire assay, which lasted for 24 h. After 24 h, the donor chambers were washed with 5 mL of PBS.

The post-assay integrity of the membranes was assessed by measuring TEWL (Table S7). After measurements, the membranes were carefully collected and placed in dry containers, and frozen at -20 °C. The receiving chambers were washed with 2 × 2.5 mL of PBS, and the solution was transferred to dry containers, and frozen at – 20 °C. The description of sample cell collection, as per the methodology, is presented in Table 2.

**Table 2:** Sample collection description

Sample Collection	Methodology
Donor chamber after 1 h of sample contact	Wash with 5 × 1mL of receiving solution. Frozen at -20 °C.
Donor chamber after 24 h	Wash with 5 mL of receiving solution. Frozen at -20 °C.
Biological membrane	Dry collection. Frozen at -20 °C.
Receiving chamber	Total camera volume receiver collection, 5 mL. Frozen at -20 °C.
Receiving chamber Wash	2 × 2.5mL receiver chamber wash. Frozen at -20 °C.

The chemical elements in the receiving solution samples were determined using Inductively Coupled Plasma-Mass Spectrometry (ICP-MS) Agilent Technologies 7700 Series. The blank sample (negative control) was prepared using PBS pH 7.4.

#### Dermal absorption estimation

Flux refers to the rate at which substances move across the membrane per unit of time (Zatz and Segers 1998). The calculation for flux involves the measurement of the substance flowing through the biological membrane and is graphically represented over time (OECD 2004b). In this study, a single time point is considered: 24 h with 1 h of peloid membrane contact, with a focus on the effects of peloid exposure. The data analysis entails comparing the results of cells exposed to peloid with those of cells not exposed to peloid, to assess the impact of peloid exposure.

The quantity of the released active substance is expressed in mass units (mg or µg) per unit of surface area and time. In cases of finite dose conditions of exposure, dermal absorption is determined using Eq. 1 (OECD 2004a).

1 $$Dermal\,absorption\equiv Skin\,washings +Skin +Receptor\,Fluid +Cell\,washings$$

The cumulative amount of the test substance (peloid) that permeated through the membrane by the end of the assay was evaluated. The permeation of chemical elements from the peloids per unit of skin surface area (A_TOTAL_, µg/cm^2^) was calculated using Eq. 2 for a membrane area of 1.77 cm^2^ (A):

2 $${A}_{TOTAL}=\frac{Dermal\,absorption}{A}$$

The assessment of the analytical data’s quality control was carried out by conducting a mass balance (Mass Balance, %) as outlined in Eq. 3. The mass balance calculation was conducted following the in vitro basic criteria outlined in the OECD guidelines and the ‘Quality of Transdermal Patches’ guideline (EMA 2014). The total recovery of chemical elements at the end of the experiment was determined by considering the initial chemical element mass of the peloid applied to the donor chamber (substance mass) and the sum of the final cumulative amount (dermal absorption, as calculated in Eq. 1). An overall recovery of the active substance within a range of 90–110% is considered acceptable without requiring justification, while any larger variation should be justified and explained (EMA 2018).

3 $$Mass Balance=\frac{Total\,recovered\,mass}{Total\,applied\,mass} \times 100$$

The results are presented as mean ± standard error of the mean (SE). The experimental data acquired for both peloids underwent analyses using an unpaired Student’s t-test to compare the values obtained in the Franz cells experiment. Additionally, a two-factor without replication was performed in Microsoft 365 Excel for data analysis. This ANOVA allowed for the comparison of variance between the two peloids. A significance threshold of *p* < 0.05 was established to determine statistically significant distinctions between the peloids.

## Results

The choice of Ca and Mg as the primary elements for permeation analysis is based on their absence in the PBS solution, as well as their biocompatibility, and potential clinical applications (Malgorzata 2019). These characteristics guarantee that the permeation measurements solely reflect the intrinsic properties of the peloids, free from any interference or influence from the PBS. The data supporting the findings of this study are available in the supplementary information in tabular form (Tables S8 to S13).

Table 3 displays the total content of the permeated chemical elements at the end of the experiment. The elements which exhibited the least variability in dermal absorption rates, along with minimal standard error values in both peloids, were Mo, Pb, Li, V and Ni. This suggests that these elements may have more reliable and consistent dermal absorption rates. Moderate variability, as indicated by standard error values that show some spread around the mean, was found in Co, Ba, Cu, Mn and Cr. On the other hand, Al, Sr, Zn, Mg, Fe, Ca showed higher variability in their dermal absorption rates, with larger standard error values, indicating a greater spread of data points around the mean.

**Table 3:** Total contents of permeated chemical elements

	Dermal absorption (mg) ± SE		Permeation (µg/cm^2^)	
	CRO peloid (*n* = 7)	CR peloid (*n* = 6)	CRO peloid	CR peloid
Al	3.72 × 10^− 1^ ± 5.38 × 10^− 2^	2.84 × 10^− 2^ ± 7.04 × 10^− 3^	210	16.04
Ba	2.21 × 10^− 3^ ± 3.35 × 10^− 4^	7.57 × 10^− 4^ ± 2.54 × 10^− 4^	1.25	0.43
Ca	1.86 × 10^0^ ± 1.40 × 10^− 1^	7.74 × 10^− 1^ ± 1.08 × 10^− 1^	1051	435
Co	2.30 × 10^− 4^ ± 3.75 × 10^− 5^	1.94 × 10^− 4^ ± 1.28 × 10^− 4^	0.13	0.11
Cr	5.13 × 10^− 3^ ± 8.72 × 10^− 4^	2.24 × 10^− 4^ ± 4.61 × 10^− 5^	2.90	0.13
Cu	1.24 × 10^− 3^ ± 3.86 × 10^− 4^	6.06 × 10^− 4^ ± 1.36 × 10^− 4^	0.70	0.34
Fe	2.99 × 10^− 1^ ± 4.22 × 10^− 2^	9.06 × 10^− 3^ ± 3.51 × 10^− 3^	169	5.12
Li	2.36 × 10^− 4^ ± 7.07 × 10^− 5^	2.27 × 10^− 5^ ± 4.74 × 10^− 6^	0.13	0.01
Mg	3.42 × 10^− 1^ ± 2.56 × 10^− 2^	2.97 × 10^− 1^ ± 2.47 × 10^− 2^	192	169
Mn	8.76 × 10^− 3^ ± 1.66 × 10^− 3^	2.29 × 10^− 3^ ± 4.97 × 10^− 4^	4.95	1.29
Mo	2.26 × 10^− 4^ ± 9.21 × 10^− 5^	6.07 × 10^− 6^ ± 3.93 × 10^− 6^	0.13	0.003
Ni	4.27 × 10^− 3^ ± 1.65 × 10^− 3^	5.99 × 10^− 5^ ± 1.32 × 10^− 5^	2.41	0.03
Pb	1.85 × 10^− 5^ ± 3.69 × 10^− 6^	1.41 × 10^− 5^ ± 3.15 × 10^− 6^	0.01	0.01
Sr	1.23 × 10^− 3^ ± 2.01 × 10^− 4^	1.47 × 10^− 3^ ± 3.20 × 10^− 4^	0.70	0.83
V	5.46 × 10^− 4^ ± 8.83 × 10^− 5^	1.20 × 10^− 4^ ± 2.59 × 10^− 5^	0.31	0.07
Zn	6.82 × 10^− 4^ ± 1.71 × 10^− 4^	1.57 × 10^− 4^ ± 5.91 × 10^− 5^	0.39	0.09

Although the differences observed in the permeation rates of the elements between the two peloids were not statistically significant *(p* > 0.05) for each peloid, it was observed that the Cró peloid exhibited higher permeation for Al, Ba, Ca, Cr, Cu, Fe, Li, Mn, Mo, Ni, V and Zn. Close permeation rates were found for Co, Mg, Pb, and Sr.

## Discussion

The Franz diffusion method is widely recognized and regulatorily accepted for assessing the systemic risk resulting from dermal exposure to chemicals. Its utility extends to serving as a valuable tool for assessing peloid formulations. However, it is important to note that diverse in vitro studies have employed Franz diffusion cells and various methodologies to investigate the transdermal delivery of constituent chemical elements found in peloids (Sánchez-Espejo et al. 2015; Khiari et al. 2019; Tateo et al. 2009).

These studies have provided valuable insights into the permeation of exchangeable cations across the membranes and the detectability of hazardous elements upon skin application. Nonetheless, it is important to acknowledge that dermal absorption of chemicals through the stratum corneum remains a complex process, particularly challenging within the context of pelotherapy (Bastos et al. 2022, 2023).

Mineral elements, when present in living tissues, are predominantly in an ionized state, dissolved within bodily fluids, as opposed to a dry mineral composition (Haftek et al. 2022). Keratinocytes and fibroplasts are structures responsible for up to 80% of ionic transport through viable skin (Denda et al. 2003; Paweloszek et al. 2016).

Tarnowska et al. (2020) conducted skin penetration studies to define skin absorption profiles of Mg^2+^ and Ca^2+^ in cosmetics using thermal spring waters (TSW) as active ingredients. The skin absorption of these cations was studied using in vitro static Franz diffusion under infinite dose conditions and with occlusion of the apparatus. Their findings indicated that the beneficial effect of TSW was primarily due to the active components, specifically calcium and magnesium, which reached viable skin layers, and that the effectiveness depended on the type of formulation.

Studies conducted in thermal waters have shown that the penetration of anions, such as Cl^−^, and monovalent cations, such as Na^+^ and K^+^, is easier compared to polyvalent ones, such as Mg^2+^ and Ca^2+^, which tend to be retained within the skin layers. The benefits of using thermal waters as active ingredients in cosmetic formulations are influenced by the skin’s exposure to cations, specifically Mg^2+^ and Ca^2+^_,_ which exert more biological functions on the skin than anions. The dependency of benefits is intricately linked to the cosmetic formulation model, impacting the absorption and distribution of these active and beneficial cations across different skin layers (Tarnowska 2020 and authors therein).

The identification and quantification of these elements within the skin depend on the analytical methodologies employed. Macroelements such as Ca, sodium (Na), potassium (K), Mg, phosphorus (P), sulfur (S), and chlorine (Cl) play pivotal roles in facilitating proper bodily functions. Sodium and K are vital for intracellular and extracellular equilibrium, while oligoelements hold specific biochemical significance within the human body (Tateo et al. 2009).

Specific trace elements found in the skin’s surface are recognized as environmental contaminants. These include elements such as arsenic (As), boron (B), chromium (Cr), nickel (Ni), strontium (St), vanadium (V), as well as aluminum (Al), zirconium (Zr), silver (Ag), gold (Au), mercury (Hg), and silicon (Si). Haftek et al. (2022) illustrated the distribution of predominant minerals within the human epidermis through a schematic drawing, based on literature data. Sodium (Na), S, K, P, Ca, Zn, and iron (Fe) were reported in relative concentrations (mg/g). Notably, quantitative data for Mg were unavailable, and copper (Cu) levels fell below the sensitivity threshold of the detection method. Calcium measurements exhibited inconsistency across studies due to differences in detection methodologies.

The evaluation of the intrinsic dissolution of the studied peloids provided valuable insights into the solubility of their elemental components after 1 h of skin contact. The conventional therapeutic mud treatments typically last 20 and 30 min. Extending the collection time to 1 h allowed for a more comprehensive assessment of element dissolution and its potential relationship with therapeutic benefits. This extended timeframe provided a broader understanding of the dissolution mechanism, including the peloid that remained on the skin, and its implications for therapeutic efficacy.

The assessment of skin permeation and estimation of the transport of constituent elements from peloid Cró and peloid Caldas da Rainha followed a standardized methodology, guaranteeing the validity and relevance of the obtained data.

The temperature of the peloid, known for its impact on therapeutic efficacy, was used as a parameter to assess the skin permeation of these chemical constituents (Carretero 2020), justification for conducting the study under finite dose conditions, and 1 h skin contact.

The mass balance results were not within the established acceptance criteria range of an overall recovery 90–110% (Table S12). This discrepancy may be attributed to several factors, including the that the experiment only had a single extraction point, variations in the test substance concentration in the peloids, the use of a frozen membrane, and the amount of peloid placed in the membrane.

Precise assessment of skin permeation was upheld through measures to control water loss via the skin. However, it was observed that several cells exhibited a significant increase in transepidermal water loss (TEWL) values post-study compared to their initial measurements. This phenomenon suggests a possible compromise in skin integrity, potentially influencing the permeation of substances.

While minor TEWL fluctuations usually align with OECD guidelines, the TEWL data obtained from this study revealed significant deviations in several assays. Notably, in assay 2 (cells 3 and 5), assay 3 (cells 1,2, 5, and 6), and assay 5, the values consistently exceeded the 10 thresholds. It is worth mentioning that negative control cells (cell 5 in assay 1 and cell 4 in assay 4) held lesser significance in the analysis (they served as benchmarks for comparison since they did not receive the test substance).

Furthermore, scrutiny of the skin revealed apparent membrane damage in specific cells. In assay 1, cell membranes 1 and 2 exhibited visible damage in the contact area, while membrane 6 showed borderline impairment. Similarly, in assay 2, evident damage was observed on cells 3 and 5 in the contact area. Cells 3, 4, and 5 retained adequate moisture on their surfaces after the removal of the donor chamber. However, gentle drying led to complete dryness, possibly contributing to the observed contact area damage.

To enhance the precision and reliability of the results, rigorous selection criteria were applied for total permeation calculations. Only cells meeting these criteria were included: initial and final TEWL levels below 10, in addition to a satisfactory visual membrane inspection (Table S6). As a result, the following were excluded: cells 1 and 2 from assay 1, cell 3 from assay 2, cells 1 and 2 from assay 3, and cell 2 from assay 5. This stringent selection process was implemented to increase the overall trustworthiness and validity of the obtained outcomes.

In terms of calcium transference, the Cró peloid (CRO) exhibited a higher total amount that had permeated the membrane, measuring 1051 µg/cm^2^. This contrasted with the peloid Caldas da Rainha (CR), which showed a comparatively lower value of 435 µg/cm^2^. Conversely, regarding magnesium, there were no significant changes in transference, with values of 192 µg/cm^2^ for CRO and 169 µg/cm^2^ for Bastos et al. (2023) explored the interaction of these peloids with artificial sweat, a simulation that emulates the sweating effect and concurrent vasodilation experienced during pelotherapy. Their investigation revealed that the extracted amount of potentially toxic elements was notably low, and in certain cases, even undetectable. However, calcium and magnesium were present in higher concentrations in the Caldas da Rainha peloid when compared with the Cró peloid.

These findings suggest that the two peloids, Cró and Caldas da Rainha, exhibit distinct properties regarding the dermal absorption of Ca and Mg. The Cró peloid appears to have a higher capacity for transporting calcium through the skin, likely due to the retention of calcium on the skin´s surface after 1 h of contact with the membrane. The outcomes related to magnesium imply that the skin did not absorb magnesium from the peloids during the experiment, which is consistent with existing information regarding magnesium dermal absorption.

Magnesium plays a pivotal role in the formation of the stratum corneum and is one of the most abundant cations within cells. Scientific interest in facilitating the transdermal movement of magnesium ions (Mg^2+^) is increasing due to its potential in promoting tissue regeneration. Several approaches can be considered to enhance the percutaneous migration of magnesium ions. Specialized devices like iontophoresis, which utilizes electric current to drive ion movement through the skin, represent one approach (Bastos et al. 2022) for controlled release for Mg^2+^ dispersion over time (Chandrasekaran et al. 2014; Liao et al. 2016; Wang et al. 2023; Yang et al. 2023).

After scrutinizing the overall permeation of trace and potentially toxic elements within the two peloids, no notable distinctions were observed between the peloids, despite differences in Al, barium (Ba), Cr, Fe, manganese (Mn), and Ni content in both peloids.

The study’s findings revealed that exchangeable cations and trace elements can permeate the membrane via diffusion, even in small amounts, suggesting that the composition of peloids plays a significant role in the diffusivity of chemical elements. It is important to acknowledge that using different membrane donors in the same assay may have contributed to variations in the results. However, accurately predicting the extent of this effect can be challenging due to factors such as donor age, skin thickness, permeability, and composition, which can vary among individuals within the same age group. To minimize this potential source of variability, future studies should aim to use membranes from donors of similar ages or, ideally, the same membrane for all assays. This approach would enhance the consistency and comparability of results across different experimental conditions.

## Conclusions

The selected experimental parameters encompass the conditions of the in vitro permeation assay of cells via the donor membrane and the methodology for sample collection. These parameters collectively elucidate the cutaneous pharmacokinetics of heated peloids upon contact with the skin. Notably, despite no apparent differences between peloids’ chemical composition, the method identified permeation variations among chemical elements. Exchangeable cations and trace elements can permeate the membrane through diffusion, even in small quantities. This suggested that the compositions of peloids significantly influences the diffusivity of chemical elements.

Meeting the stringent 90–110% recovery criteria for peloid chemical element balance outlined by OECD guidelines presents a notable challenge. Nonetheless, measurements to assess skin integrity before and after the Franz diffusion experiment revealed the well-preserved overall barrier function and integrity of the skin.

The methodology employed in this study is reproducible and holds the potential for application in the analysis of pre-formulation peloids. Furthermore, it provides a means to evaluate the performance of therapeutic elements during topical administration, including those with potential toxicity concerns.

The outcomes of the study emphasize the potential transport properties of chemical elements within the two types of peloids. This underscores the significance of careful selection when choosing peloids for specific applications or therapeutic purposes. Multiple factors influence the transdermal delivery and absorption of peloid elements, including the physicochemical attributes of peloid constituents, formulation characteristics, skin condition, and the presence of coexisting substances. A profound understanding of these intricacies is essential for optimizing peloid formulations, ensuring their safety and efficacy in pelotherapy.

By considering these factors and adopting the validated methodology presented herein, further avenues of research and development can be pursued to advance the understanding and utilization of peloids in therapeutic applications. This endeavour stands to refine treatments and guarantee the prudent and efficient utilization of peloids within spa settings.

### Electronic supplementary material

Below is the link to the electronic supplementary material.

Supplementary Material 1

## References

[CR2] Bastos CM, Rocha F (2022). Assessment of some clay-based products available on market and designed for topical use. J Geosci.

[CR3] Bastos CM, Rocha F (2023) Experimental peloid formulation using a Portuguese bentonite and different mineral-medicinal waters suitable for therapeutic and well-being purposes. Clays Clay Miner. 10.1007/s42860-023-00260-6

[CR4] Bastos CM, Rocha F, Gomes N, Marinho-Reis P (2022). The challenge in combining Pelotherapy and Electrotherapy (Iontophoresis) in one single therapeutic modality. Appl Sci.

[CR1] Bastos CM, Rocha F, Patinha C, Marinho-Reis P (2023). Bioaccessibility by perspiration uptake of minerals from two different sulfurous peloids. Environ Geochem Health.

[CR5] Cao L, Xie W, Cui H, Xiong Z, Tang Y, Zhang X, Feng Y (2022). Fibrous Clays in Dermopharmaceutical and Cosmetic Applications: traditional and emerging perspectives. Int J Pharm.

[CR7] Carretero MI (2002) Clay minerals and their beneficial effects upon human health. A review. Appl. Sci. *21 (3–4)* 155–163. 10.1016/S0169-1317(01)00085-0

[CR9] Carretero MI (2020). Clays in pelotherapy. A review. Part I: Mineralogy, chemistry, physical and physicochemical properties. Appl Sci.

[CR6] Carretero MI, Pozo M (2009). Clay and non-clay minerals in the pharmaceutical industry. Part I. excipients and medical applications. Appl Sci.

[CR8] Carretero MI, Pozo M, Martín-Rubí JA, Pozo E, Maraver F (2010). Mobility of elements in interaction between artificial sweat and peloids used in Spanish spas. Appl Sci.

[CR10] Chandrasekaran NC, Weir C, Alfraji S, Grice J, Roberts MS, Barnard RT (2014). Effects of magnesium deficiency – more than skin deep. Exp Biol Med.

[CR11] Denda M, Fuziwara S, Inoue K (2003). Influx of calcium and chloride ions into epidermal keratinocytes regulates exocytosis of epidermal lamellar bodies and skin permeability barrier 73 homeostasis. J Invest Dermatol.

[CR13] European Medicines Agency (EMA) (2018) Draft guideline on quality and equivalence of topical products. CHMP/QWP/708282/2018

[CR12] European Medicines Agency (EMA) (2014) Guideline on quality of transdermal patches – Scientific guideline. Committee for Medicinal Products for Human Use (CHMP). EMA/CHMP/QWP/608924/2014

[CR14] Haftek M, Abdayem R, Guyonnet-Debersac P (2022). Skin minerals: key roles of inorganic elements in skin physiological functions. Int J Mol Sci.

[CR15] Khiari I, Sánchez-Espejo R, García-Villén F, Cerezo P, Aguzzi C, López-Galindo A, Jamoussi F, Viseras C (2019). Rheology and cation release of Tunisian medina mud-packs intended for topical applications. Appl Sci.

[CR16] Kovács A, Zsikó S, Falusi F, Csányi E, Budai-Szűcs M, Csóka I, Berkó S (2021). Comparison of synthetic membranes to heat-separated human epidermis in skin permeation studies in Vitro. Pharmaceutics.

[CR17] Liao A-H, Lu Y-J, Hung C-R, Yang M-Y (2016). Efficacy of transdermal magnesium ascorbyl phosphate delivery after ultrasound treatment with microbubbles in gel-type surrounding medium in mice. Mater Sci Eng C.

[CR18] Malgorzata T (2019) Evaluation of skin absorption of inorganic ions with regard to their physicochemical properties. Pharmaceutical sciences. Université de Lyon, English. (CNNT: 2019LYSE1262). https://theses.hal.science/tel-02887094

[CR19] Massaro M, Colletti CG, Lazzara G, Riela S (2018). The Use of some Clay minerals as Natural resources for drug carrier applications. J Funct Biomater.

[CR20] Metshein M, Tuulik V-R, Tuulik V, Kumm M, Min M, Annus P (2023). Electrical Bioimpedance Analysis for evaluating the effect of Pelotherapy on the human skin: methodology and experiments. Sensors.

[CR21] Muñoz MS, Rodríguez CM, Rudnikas AG, Rizo OD, Martínez-Santos M, Ruiz-Romera E, Castillo JRF, Pérez-Gramatges A, Martínez-Villegas NV, Padilla DB, Díaz RH, González-Hernández P (2015). Physicochemical characterization, elemental speciation and hydrogeochemical modeling of river and peloid sediments used for therapeutic uses. Appl Sci.

[CR22] Nomicisio C, Ruggeri M, Bianchi E, Vigani B, Valentino C, Aguzzi C, Viseras C, Rossi S, Sandri G (2023). Natural and Synthetic Clay Minerals in the Pharmaceutical and Biomedical Fields. Pharmaceutics.

[CR24] OECD (2004). Test No. 428: skin absorption. Vitro Method, OECD guidelines for the testing of Chemicals.

[CR23] OECD (2004). Guidance Document for the Conduct of skin absorption studies. OECD series on testing ad Assessment., No. 28.

[CR25] OECD (2011) Guidance Notes on Dermal Absorption (GN 156). Series on Testing and Assessment, OECD Publishing, 18 August 2011

[CR26] Paweloszek R, Briançon S, Chevalier Y, Gilon-Delepine N, Pelletier J, Bolzinger MA (2016). Skin absorption of anions: part two. Skin absorption of halide ions. Pharm Res.

[CR27] Sánchez-Espejo R, Cerezo P, Aguzzi C, López-Galindo A, Machado J, Viseras C (2015). Physicochemical and *in vitro* cation release relevance of therapeutic muds maturation. Appl Clay Sci.

[CR28] SCCS (Scientific Committee on Consumer Safety) (2010) European Commission, Directorate-General for Health and Consumers. Basic criteria for the in vitro assessment of dermal absorption of cosmetic ingredients. https://data.europa.eu/doi/10.2772/25843

[CR29] Shah VP, Yacobi A, Rădulescu FŞ, Miron DS, Lane ME (2015). A science based approach to topical drug classification system (TCS). Int J Pharm.

[CR32] Tarnowska M, Briançon S, Resende de Azevedo J, Chevalier Y, Arquier D, Barratier C, Bolzinger M-A (2020). The effect of vehicle on skin absorption of Mg^2+^ and Ca^2+^ from thermal spring water. Int J Cosmet Sci.

[CR30] Tateo F, Summa V (2007). Element mobility in clays for healing use. Appl Sci.

[CR31] Tateo F, Ravaglioli A, Andreoli C, Bonina F, Coiro V, Degetto S, Giarreta A, Orsini AM, Puglia C, Summa V (2009). The in-vitro percutaneous migration of chemical elements from a thermal mud for healing use. Appl Sci.

[CR33] Tian X, Zhang Y, Li H, Jiao Y, Wang Q, Zhang Y, Ma N, Wang W (2022). Property of mud and its application in cosmetic and medical fields: a review. Environ Geochem Health.

[CR34] United States Pharmacopeia (USP) (2014) General Chapter, 〈1724〉 Semisolid Drug products—performance tests. USP 37 1273–1284. 10.31003/USPNF_M5695_01_01

[CR35] Viseras C, Carazo E, Borrego-Sánchez A, García-Villén F, Sánchez-Espejo R, Cerezo P, Aguzzi C (2019). Clay minerals in skin drug delivery. Clays Clay Min.

[CR36] Wang P, Wu J, Yang H, Liu H, Yao T, Liu C, Gong Y, Wang M, Ji G, Huang P, Wang X (2023). Intelligent microneedle patch with prolonged local release of hydrogen and magnesium ions for diabetic wound healing. Bioact Mater.

[CR37] Yang F, Xue Y, Wang F, Guo D, He Y, Zhao X, Yan F, Xu Y, Xia D, Liu Y (2023). Sustained release of magnesium and zinc ions synergistically accelerates wound healing. Bioact Mater.

[CR38] Zatz JL, Segers JD (1998). Techniques for measuring *in vitro* release from semisolids. Dissolution Tecnol.

