# Supplementary material for: Characterization of percutaneous absorption of calcium, magnesium, and potentially toxic elements in two tailored sulfurous therapeutic peloids: a comprehensive in vitro pilot study
Source: Int J Biometeorol. 2024 Mar 1;68(6):1061–72. doi: 10.1007/s00484-024-02644-2 (PMC11108904; doi:10.1007/s00484-024-02644-2)
Supplement: Supplementary file 1 — Supplementary Material 1 [file 484_2024_2644_MOESM1_ESM.docx]

**Characterization of percutaneous absorption of calcium, magnesium, and potentially toxic elements in two tailored sulfurous therapeutic peloids: a comprehensive *in vitro* pilot study**

**International Journal of Biometeorology**

**Carla Marina Bastos ^1,2*^, Fernando Rocha ^1^, Carla Patinha^1^ and Paula Marinho-Reis^3^**

^1^Department of Geosciences, GeoBioTec Research Centre, University of Aveiro, 3810-193 Aveiro, Portugal.

^2^Exatronic, Lda., 3800-373 Aveiro, Portugal.

^3^ Institute of Earth Sciences (ICT) – Pole of the University of Minho, University of Minho, 4710-057 Braga, Portugal;

*Corresponding author: mbastos@exatronic.pt

**Table S1.** Primary chemical components of the maturation matrix

| **Bentonite** | | **Mineral-medicinal water** | | |
| --- | --- | --- | --- | --- |
| Benavila | |  | Cró | Caldas da Rainha |
| Br (ppm) | 1.1 | Ag (µg/L) | 0.53 | - |
| Ce (ppm) | 31.4 | Al (µg/L) | 6.4 | 13 |
| Co (ppm) | 21.2 | As (µg/L) | 12.8 | 6 |
| Cs (ppm) | 6.1 | B (µg/L) | - | < d.l |
| Cu (ppm) | 93.7 | Ba (µg/L) | <1.3 | 38 |
| Ga (ppm) | 9 | Be (µg/L) | 0.60 | - |
| Mo (ppm) | 1.1 | Bi (µg/L) | - | - |
| Nb (ppm) | 2.9 | Ca (mg/L) | 3.9 | 305 |
| Ni (ppm) | 250 | Cd (µg/L) | 0.22 | - |
| Pb (ppm) | 2.7 | Co (µg/L) | <0.36 | - |
| Rb (ppm) | 24.7 | Cr (µg/L) | <2.1 | 21 |
| Sc (ppm) | 43 | Cu (µg/L) | <1.1 | 4 |
| Sn (ppm) | 4.3 | Fe (µg/L) | 26 | 5 |
| Sr (ppm) | 67.8 | K (mg/L) | 2.8 | 5 |
| U (ppm) | 1.4 | Li (µg/L) | 675 | 67 |
| Y (ppm) | 11 | Mg (mg/L) | 0.2 | 61 |
| W (ppm) | 0 | Mo (µg/L) | 1.1 | 0.05 |
| Zn (ppm) | 62 | Mn (µg/L) | 21.1 | 19 |
| Zr (ppm) | 46.7 | Na (mg/L) | 98.7 | 662 |
| SiO_2_ (wt.%) | 35.10 | Ni (µg/L) | <17.2 | - |
| Al_2_O_3_ (wt.%) | 8.87 | P (µg/L) | - | - |
| Fe_2_O_3_ (wt.%) | 14.21 | Pb (µg/L) | <0.65 | - |
| CaO (wt.%) | 19.39 | Sb (µg/L) | <0.14 | - |
| TiO_2_ (wt.%) | 0.785 | Sn (µg/L) | <0.47 | - |
| MnO (wt.%) | 0.069 | Sr (µg/L) | - | - |
| K_2_O (wt.%) | 0.467 | Th (µg/L) | - | 13 |
| P_2_O_5_ (wt.%) | 0.025 | Tl (µg/L) | <0.21 | 6 |
| MgO (wt.%) | 6.33 | U (µg/L) | - | < d.l |
| Na_2_O (wt.%) | 0.071 | V (µg/L) | <0.49 | 38 |
| SO_3_ (wt.%) | 0.007 | W (µg/L) | 76.9 | - |
|  |  | Zn (µg/L) | <6.2 | - |

**Table S2.** Skin donor’s information

| **Test System code - (Skin)** | **Donor age** | **Peloid Assay** |
| --- | --- | --- |
| LF0020-MA | 51 | CRO |
| LF0020-PD | 40 |  |
| LF0020-CT | 25 |  |
| LF0020-LASMM | 36 | CR |
| LF0020-CIPS | 32 |  |
| LF0020-SMRP | 41 |  |

**Table S3.** Equipment and materials.

| Diffusion cell system | Franz SES GMBH, Model V6A-02, with shaking rack for 6 cells, 5 mL volume (receptor chamber - basal), 15 mm diameter, exposure area:1.77 cm2.  System coupled to a thermoregulated circulation bath at 32℃ (±1), Thermo Scientific, Model Haake SC100. |
| --- | --- |
| Weighing machine | Analytic: VWR, Model LA254i  Precision: VWR, Model SE1202 |
| Microplate shaker | VWR, Model 444.01770 |
| Micropipettes | P5000VWR, Model VE5000; P1000, VWR, Model VE1000 |
| Digital chronometer | VWR |
| pH indicator strips | Range 6.5-10, MColorpHastTM, Merck |
| Thermoregulated immersion bath | VWB 26, VWR |
| Bath | Able to thermostatize at 45ºC and 60℃ |
| Digital thermometer | -50℃ to 300℃, Enzymatic |
| Fridge | Liebherr, Lkexv 5400 Index20B/001 |
| Freezer | FT190, electric |
| Dataloggers | Ebro, EBI20 |
| Magnifying glass | Coupled to the lamp |
| Determining device of TEWL*  (Condenser chamber) | Aquaflux, AF200, Biox, UK |
| Usual | Lab material |

*TEWL-Transepidermal water loss

**Table S4.**  Chemical composition of the PBS pH 7.4 and Quality control

| **Analyte** | **Unit** | **Detection Limit** | **PBS pH 7.4** | **Quality Control** |
| --- | --- | --- | --- | --- |
| Na | mg/L | 0.50 | 5,079.4 | 3,913.2 |
| P | mg/L | 1.00 | 425,599.3 | 360,988.5 |
| K | µg/L | 0.50 | 249.8 | 221.34 |
| Cr | µg/L | 0.10 | 1.69 | 1.33 |
| Rb | µg/L | 60.0 | 2.54 | 2.86 |
| Sr | µg/L | 1.50 | 4.69 | 7.10 |
| Mo | µg/L | 0.50 | 0.60 | 0.99 |
| Cd | µg/L | 1.00 | 5.31 | < d.l. |

**Table S5.** Peloid sample mass (g) added to each cell.

|  | **Cell 1** | **Cell 2** | **Cell 3** | **Cell 4** | **Cell 5** | **Cell 6** |
| --- | --- | --- | --- | --- | --- | --- |
| Assay 1 | 0.0357 | 0.0340 | --- | 0.0529 | 0.0471 | 0.045 |
| Assay 2 | 0.0364 | 0.0494 | 0.0483 | 0.0442 | --- | --- |
| Assay 3 | 0.0348 | 0.0431 | 0.0407 | 0.0421 | --- | 0.0512 |
| Assay 4 |  | 0.0585 | 0.0549 | --- |  |  |
| Assay 5 |  | 0.0503 | 0.0543 | --- |  |  |

**Table S6.** Visual inspection of membrane integrity at the end of the tests performed (C - Compliant; NC – Noncompliant)

| **Cell 1** | **Cell 2** | **Cell 3** | **Cell 4** | **Cell 5** | **Cell 6** |
| --- | --- | --- | --- | --- | --- |
| **Assay 1** | | | | | |
| NC | NC | C | C | C | NC |
| **Assay 2** | | | | | |
| C | C | NC | C | NC | C |
| **Assay 3** | | | | | |
| C | C | C | C | C | C |
| **Assay 4** | | | | | |
| --- | C | C | C | --- | --- |
| **Assay 5** | | | | | |
| --- | C | C | C | --- | --- |

**Table S7.** TEWL (g/m^2^h) recorded before sample application (t_1h,_ average of measurements n=2) and end of study (t_24h_)

|  | **Cell 1** | **Cell 2** | **Cell 3** | **Cell 4** | **Cell 5** | **Cell 6** |
| --- | --- | --- | --- | --- | --- | --- |
| **Assay 1** | | | | | | |
| **t_1h_** | 8.77 | 13.56 | 12.55 | 8.11 | 8.28 | 8.62 |
| **t_24h_** | 10.17 | 11.76 | 5.53 | 4.82 | 10.85 | 7.28 |
| **Assay 2** | | | | | | |
| **t_1h_** | 6.68 | 9.61 | 8.91 | 10.05 | 12.54 | 8.99 |
| **t_24h_** | 6.62 | 6.90 | 17.43 | 12.17 | 15.59 | 8.69 |
| **Assay 3** | | | | | | |
| **t_1h_** | 17.44 | 15.62 | 11.17 | 11.07 | 8.69 | 8.33 |
| **t_24h_** | 20.42 | 11.05 | 9.14 | 8.43 | 18.22 | 11.48 |
| **Assay 4** | | | | | | |
| **t_1h_** | --- | 15.37 | 20.31 | 20.87 | --- | --- |
| **t_24h_** | --- | 9.57 | 9.59 | 12.70 | --- | --- |
| **Assay 5** | | | | | | |
| **t_1h_** | --- | 12.74 | 11.14 | 13.77 | --- | --- |
| **t_24h_** | --- | 13.16 | 9.49 | 9.43 | --- | --- |

**Table S8.**  Cró (Assay 1 and Assay 2) - essential elements (Ca and Mg) concentration by ICP-MS.

| **Assay 1** | **Mg** | **Ca** | **Assay 2** | **Mg** | **Ca** |
| --- | --- | --- | --- | --- | --- |
| Donor chamber washing after 1 h sample contact (mg/L) | | | | | |
| Cell 1 | 56 | 230 | Cell 1 | 59 | 252 |
| Cell 2 | 71 | 288 | Cell 2 | 62 | 281 |
| Cell 3* | < d.l. | 0.50 | Cell 3 | 59 | 268 |
| Cell 4 | 82 | 356 | Cell 4 | 68 | 286 |
| Cell 5 | 58 | 252 | Cell 5* | < d.l. | 0.69 |
| Cell 6 | 58 | 251 | Cell 6* | < d.l. | 0.50 |
| Donor chamber after 24 h (mg/L) | | | | | |
| Cell 1 | 1.92 | 7.10 | Cell 1 | < d.l. | 1.52 |
| Cell 2 | < d.l. | 0.72 | Cell 2 | < d.l. | 1.06 |
| Cell 3* | < d.l. | 0.64 | Cell 3 | < d.l. | 0.83 |
| Cell 4 | 0.13 | 1.41 | Cell 4 | < d.l. | 1.17 |
| Cell 5 | < d.l. | 0.88 | Cell 5* | < d.l. | 0.94 |
| Cell 6 | < d.l. | 1.21 | Cell 6* | < d.l. | 1.75 |
| Receiving chamber (mg/L) | | | | | |
| Cell 1 | < d.l. | 1.42 | Cell 1 | < d.l. | 0.51 |
| Cell 2 | 0.18 | 5.12 | Cell 2 | < d.l. | 0.56 |
| Cell 3* | < d.l. | 0.81 | Cell 3 | < d.l. | 0.80 |
| Cell 4 | < d.l. | 0.66 | Cell 4 | < d.l. | 0.72 |
| Cell 5 | 0.10 | 9.48 | Cell 5* | < d.l. | 0.67 |
| Cell 6 | < d.l. | 0.74 | Cell 6* | < d.l. | 0.88 |
| Receiving chamber Wash (mg/L) | | | | | |
| Cell 1 | < d.l. | 0.00 | Cell 1 | 0.15 | 1.15 |
| Cell 2 | < d.l. | 0.58 | Cell 2 | 0.16 | 1.23 |
| Cell 3* | < d.l. | 0.00 | Cell 3 | < d.l. | 0.00 |
| Cell 4 | < d.l. | 0.31 | Cell 4 | < d.l. | 0.52 |
| Cell 5 | < d.l. | 4.51 | Cell 5* | < d.l. | 0.38 |
| Cell 6 | < d.l. | 0.00 | Cell 6* | < d.l. | 0.00 |
| **Assay 1** | **Mg** | **Ca** | **Assay 2** | **Mg** | **Ca** |
| Biological membrane (mg/Kg) | | | | | |
| Cell 1 | 96.30 | 1215 | Cell 1 | 86.0 | 849 |
| Cell 2 | 75.00 | 581 | Cell 2 | 109 | 1249 |
| Cell 3* | 54.43 | 405 | Cell 3 | 108 | 828 |
| Cell 4 | 137.31 | 2,116 | Cell 4 | 104 | 587 |
| Cell 5 | 82.22 | 746 | Cell 5* | 107 | 523 |
| Cell 6 | 81.60 | 824 | Cell 6* | 134 | 615 |

*Blank sample. d.l. – detection limit.

**Table S9.**  Caldas da Rainha (Assay 3, 4 and Assay 5) – essential elements (Ca and Mg) concentration by ICP-MS.

| **Assay 3** | **Mg** | **Ca** | **Assay 4** | **Mg** | **Ca** | **Assay 5** | **Mg** | **Ca** |
| --- | --- | --- | --- | --- | --- | --- | --- | --- |
| Donor chamber washing after 1 h sample contact (mg/L) | | | | | | | | |
| Cell 1 | 27 | 166 | Cell 2 | 58 | 192 | Cell 2 | 53 | 219 |
| Cell 2 | 31 | 107 | Cell 3 | 51 | 109 | Cell 3 | 51 | 82 |
| Cell 3 | 33 | 177 | Cell 4* | < d.l. | 0.44 | Cell 4* | < d.l. | < d.l. |
| Cell 4 | 42 | 111 |  |  |  |  |  |  |
| Cell 5* | < d.l. | 0.55 |  |  |  |  |  |  |
| Cell 6 | 47 | 170 |  |  |  |  |  |  |
| Donor chamber after 24 h (mg/L) | | | | | | | | |
| Cell 1 | < d.l. | 1.43 | Cell 2 | 0.75 | 5.90 | Cell 2 | < d.l. | 0.92 |
| Cell 2 | < d.l. | 1.42 | Cell 3 | < d.l. | 0.94 | Cell 3 | < d.l. | 0.54 |
| Cell 3 | < d.l. | 0.85 | Cell 4* | < d.l. | 0.70 | Cell 4* | < d.l. | 0.34 |
| Cell 4 | < d.l. | 2.14 |  |  |  |  |  |  |
| Cell 5* | < d.l. | 0.78 |  |  |  |  |  |  |
| Cell 6 | < d.l. | 1.49 |  |  |  |  |  |  |
| Receiving chamber (mg/L) | | | | | | | | |
| Cell 1 | < d.l. | 0.90 | Cell 2 | 0.19 | 1.67 | Cell 2 | 0.21 | 1.94 |
| Cell 2 | < d.l. | 0.83 | Cell 3 | 0.65 | 6.05 | Cell 3 | 0.18 | 1.16 |
| Cell 3 | < d.l. | 0.87 | Cell 4* | 0.12 | 1.11 | Cell 4* | 0.20 | 1.31 |
| Cell 4 | < d.l. | 0.92 |  |  |  |  |  |  |
| Cell 5* | < d.l. | 1.99 |  |  |  |  |  |  |
| Cell 6 | < d.l. | 0.78 |  |  |  |  |  |  |
| Receiving chamber Wash (mg/L) | | | | | | | | |
| Cell 1 | < d.l. | 0.50 | Cell 2 | < d.l. | 0.64 | Cell 2 | < d.l. | 0.45 |
| Cell 2 | < d.l. | 0.48 | Cell 3 | < d.l. | 0.67 | Cell 3 | < d.l. | 0.33 |
| Cell 3 | < d.l. | 0.52 | Cell 4* | < d.l. | 0.46 | Cell 4* | < d.l. | < d.l. |
| Cell 4 | < d.l. | 0.49 |  |  |  |  |  |  |
| Cell 5* | < d.l. | 0.52 |  |  |  |  |  |  |
| Cell 6 | < d.l. | 0.47 |  |  |  |  |  |  |
| **Assay 3** | **Mg** | **Ca** | **Assay 4** | **Mg** | **Ca** | **Assay 5** | **Mg** | **Ca** |
| Biological membrane (mg/Kg) | | | | | | | | |
| Cell 1 | 844 | 375 | Cell 2 | 81 | 616 | Cell 2 | 14 | < d.l. |
| Cell 2 | 543 | 256 | Cell 3 | 64 | 412 | Cell 3 | 42 | 0.146 |
| Cell 3 | 434 | 134 | Cell 4* | 82 | 473 | Cell 4* | 9 | < d.l. |
| Cell 4 | 540 | 230 |  |  |  |  |  |  |
| Cell 5* | 387 | 106 |  |  |  |  |  |  |
| Cell 6 | 521 | 157 |  |  |  |  |  |  |

*Blank sample. d.l. – detection limit.

**Table S10.** Cró (Assay 1 and Assay 2) *–* Trace elements concentration by ICP-MS. (*Blank sample. d.l. – detection limit.)

| **Assay 1** | **Al** | **Ba** | **Co** | **Cr** | **Cu** | **Fe** | **Li** | **Mn** | **Mo** | **Ni** | **Pb** | **Rb** | **Sr** | **V** | **Zn** |
| --- | --- | --- | --- | --- | --- | --- | --- | --- | --- | --- | --- | --- | --- | --- | --- |
| **Donor chamber washing after 1 h sample contact (µg/L)** | | | | | | | | | | | | | | | |
| Cell 1 | 68,413.2 | 412.7 | 45.5 | 827.4 | 77.2 | 53,219.7 | 89.9 | 1,398.7 | 0.96 | 115.6 | 1.50 | 12.6 | 233.7 | 110.6 | 71.5 |
| Cell 2 | 84,451.0 | 525.3 | 57.4 | 1,001.3 | 100.9 | 69,171.4 | 60.5 | 1,772.4 | 0.97 | 145.8 | 4.37 | 15.6 | 300.6 | 138.9 | 94.1 |
| Cell 3* | 90.8 | < d.l. | < d.l. | < d.l. | 6.20 | 123.6 | 5.05 | < d.l. | 1.21 | < d.l. | < d.l. | 4.26 | 2.44 | < d.l. | < d.l. |
| Cell 4 | 83,748.9 | 592.2 | 69.8 | 982.2 | 116.2 | 62,506.2 | 77.8 | 2,179.2 | 2.30 | 161.7 | 5.67 | 16.1 | 376.3 | 156.9 | 0.64 |
| Cell 5 | 68,192.3 | 452.5 | 48.4 | 820.1 | 82.7 | 52,775.3 | 40.8 | 1,491. | 0.63 | 119.2 | 5.22 | 12.1 | 258.4 | 115.6 | 255.6 |
| Cell 6 | 65,983.5 | 11.0 | 45.9 | 769.3 | 76.8 | 49,641.9 | 51.4 | 1,432.0 | 0.59 | 111.5 | 3.95 | 10.41 | 260.66 | 107.3 | 0.36 |
| **Donor chamber after 24 h (µg/L)** | | | | | | | | | | | | | | | |
| Cell 1 | 4,422.6 | 22.1 | < d.l. | 55.4 | 18.7 | 3,806.6 | 5.06 | 35.80 | 1.89 | 7.66 | 4.30 | 5.31 | 8.21 | 4.89 | < d.l. |
| Cell 2 | 65.7 | 12.5 | < d.l. | 0.91 | 12.9 | 61.8 | 2.90 | < d.l. | 0.94 | 1.03 | < d.l. | 4.01 | 2.25 | < d.l. | < d.l. |
| Cell 3* | 46.3 | < d.l. | < d.l. | < d.l. | 17.4 | 31.8 | < d.l. | < d.l. | 1.72 | < d.l. | < d.l. | 4.38 | 2.57 | < d.l. | < d.l. |
| Cell 4 | 298.7 | 11.0 | < d.l. | 4.30 | 11.6 | 255.4 | 2.12 | < d.l. | 1.02 | 1.29 | < d.l. | 4.54 | 2.89 | < d.l. | 0.16 |
| Cell 5 | 197.5 | 11.9 | < d.l. | 2.2 | 11.9 | 153.6 | 2.17 | < d.l. | 0.91 | 2.41 | < d.l. | 4.54 | 4.18 | < d.l. | < d.l. |
| Cell 6 | 193.1 | 438.3 | < d.l. | 2.6 | 10.4 | 155.7 | 3.11 | < d.l. | 3.60 | 1.60 | < d.l. | 4.03 | 3.50 | < d.l. | 0.14 |
| **Receiving chamber (µg/L)** | | | | | | | | | | | | | | | |
| Cell 1 | 26.8 | 10.1 | < d.l. | 0.85 | 3.86 | 19.5 | < d.l. | < d.l. | 1.64 | < d.l. | < d.l. | 4.05 | 2.12 | < d.l. | < d.l. |
| Cell 2 | 42.7 | 10.4 | < d.l. | 1.77 | 2.26 | 45.7 | < d.l. | 4.33 | 1.09 | < d.l. | < d.l. | 4.17 | 4.72 | < d.l. | < d.l. |
| Cell 3* | 21.6 | < d.l. | < d.l. | < d.l. | 11.8 | 20.4 | < d.l. | < d.l. | 1.00 | < d.l. | < d.l. | 4.09 | 2.01 | < d.l. | < d.l. |
| Cell 4 | 25.9 | 9.80 | < d.l. | < d.l. | 5.8 | 12.1 | < d.l. | < d.l. | 0.98 | < d.l. | < d.l. | 4.14 | 1.59 | < d.l. | < d.l. |
| Cell 5 | 26.8 | 10.1 | < d.l. | 0.85 | 3.86 | 19.5 | < d.l. | < d.l. | 1.64 | < d.l. | < d.l. | 4.05 | 2.12 | < d.l. | < d.l. |
| Cell 6 | 42.7 | 10.4 | < d.l. | 1.77 | 2.26 | 45.7 | < d.l. | 4.33 | 1.09 | < d.l. | < d.l. | 4.17 | 4.72 | < d.l. | < d.l. |
| **Receiving chamber Wash (µg/L)** | | | | | | | | | | | | | | | |
| Cell 1 | < d.l. | < d.l. | < d.l. | < d.l. | 7.94 | 12.8 | < d.l. | < d.l. | 1.59 | < d.l. | < d.l. | 3.10 | < d.l. | < d.l. | < d.l. |
| Cell 2 | < d.l. | < d.l. | < d.l. | < d.l. | 10.4 | < d.l. | < d.l. | < d.l. | 0.78 | < d.l. | < d.l. | 3.06 | 1.48 | < d.l. | < d.l. |
| Cell 3* | < d.l. | < d.l. | < d.l. | < d.l. | 8.80 | 13.4 | < d.l. | < d.l. | 0.75 | < d.l. | < d.l. | 3.06 | < d.l. | < d.l. | < d.l. |
| Cell 4 | < d.l. | < d.l. | < d.l. | < d.l. | 2.0 | < d.l. | < d.l. | < d.l. | 0.77 | < d.l. | < d.l. | 2.97 | < d.l. | < d.l. | < d.l. |
| Cell 5 | < d.l. | < d.l. | < d.l. | < d.l. | 4.7 | < d.l. | < d.l. | < d.l. | 1.72 | < d.l. | < d.l. | 2.91 | 2.29 | < d.l. | < d.l. |
| Cell 6 | < d.l. | < d.l. | < d.l. | < d.l. | 1.4 | < d.l. | < d.l. | < d.l. | 0.73 | < d.l. | < d.l. | 2.90 | 1.35 | < d.l. | < d.l. |
| **Biological membrane (mg/Kg)** | | | | | | | | | | | | | | | |
| Cell 1 | 46.3 | < d.l. | < d.l | 3.40 | 116.2 | 98.1 | 0.16 | 1.32 | 0.51 | 6.49 | < d.l. | 0.85 | 0.50 | < d.l. | 180.2 |
| Cell 2 | 56.0 | 468.2 | < d.l | 1.27 | 10.7 | 72.3 | 0.17 | < d.l | 0.51 | 4.04 | < d.l. | 0.95 | < d.l. | < d.l. | 66.7 |
| Cell 3* | 28.6 | 0.70 | < d.l | 1.40 | 38.9 | 860.7 | 0.32 | 4.81 | 1.00 | 8.70 | < d.l. | 1.00 | < d.l. | < d.l. | < d.l. |
| Cell 4 | 99.0 | 0.50 | < d.l | 1.90 | 13.3 | 136.8 | 0.18 | 4.70 | 0.50 | 2.70 | < d.l. | 0.90 | 0.70 | < d.l. | 0.0007 |
| Cell 5 | 45.6 | < d.l. | < d.l | 1.08 | 30.9 | 70.1 | < d.l. | 3.54 | < d.l. | 12.0 | < d.l. | 0.94 | < d.l. | < d.l. | 61.4 |
| Cell 6 | 92.0 | < d.l. | < d.l | 0.80 | 17.5 | 63.7 | < d.l. | 1.69 | 0.40 | 3.70 | < d.l. | 1.00 | 0.5 | < d.l. | 0.0008 |

| **Assay 2** | **Al** | **Ba** | **Co** | **Cr** | **Cu** | **Fe** | **Li** | **Mn** | **Mo** | **Ni** | **Pb** | **Rb** | **Sr** | **V** | **Zn** |
| --- | --- | --- | --- | --- | --- | --- | --- | --- | --- | --- | --- | --- | --- | --- | --- |
| **Donor chamber washing after 1 h sample contact (µg/L)** | | | | | | | | | | | | | | | |
| Cell 1 | 71,621.8 | 485.7 | 49.9 | 883.2 | 79.7 | 58,240.1 | 45.2 | 1,614.5 | 0.56 | 121.2 | 4.36 | 13.3 | 256.6 | 120.5 | 75.4 |
| Cell 2 | 63,631.7 | 512.4 | 52.9 | 761.04 | 82.5 | 46,212.1 | 46.7 | 1,692.8 | < d.l. | 116.6 | 4.79 | 10.4 | 293.8 | 114.7 | 69.2 |
| Cell 3 | 60,960.2 | 503.5 | 50.7 | 656.6 | 80.0 | 43,616.9 | 54.7 | 1,602.2 | < d.l. | 115.3 | 5.04 | 12.1 | 287.7 | 110.8 | 59.5 |
| Cell 4 | 80,594.5 | 531.9 | 57.0 | 982.1 | 92.5 | 62,157.2 | 47.7 | 1,724.0 | < d.l. | 147.2 | 4.67 | 11.73 | 303.1 | 142.3 | 87.2 |
| Cell 5* | 16.9 | < d.l. | < d.l. | 1.13 | < d.l. | 118.5 | 3.89 | < d.l. | 0.96 | < d.l. | < d.l. | 4.37 | 3.06 | < d.l. | < d.l. |
| Cell 6* | 81.4 | < d.l. | < d.l. | 0.50 | < d.l. | 54.5 | 4.00 | < d.l. | 0.90 | < d.l. | < d.l. | 4.20 | 2.94 | < d.l. | < d.l. |
| **Donor chamber after 24 h (µg/L)** | | | | | | | | | | | | | | | |
| Cell 1 | 107.7 | 10.4 | < d.l. | 2.10 | 2.17 | 134.8 | < d.l. | < d.l. | 2.48 | < d.l. | < d.l. | 3.35 | 2.21 | < d.l. | 10.2 |
| Cell 2 | 28.0 | 10.2 | < d.l. | 0.57 | 2.22 | 26.8 | < d.l. | < d.l. | 3.66 | < d.l. | < d.l. | 3.24 | 1.85 | < d.l. | 11.9 |
| Cell 3 | 48.4 | 10.4 | < d.l. | 1.07 | 2.73 | 77.2 | < d.l. | < d.l. | 4.65 | < d.l. | < d.l. | 3.26 | 1.86 | < d.l. | < d.l. |
| Cell 4 | 41.5 | 11.1 | < d.l. | 1.08 | 1.60 | 88.6 | < d.l. | < d.l. | 2.65 | < d.l. | < d.l. | 3.34 | 2.18 | < d.l. | 12.7 |
| Cell 5* | < d.l. | < d.l. | < d.l. | < d.l. | < d.l. | 33.0 | < d.l. | < d.l. | 2.34 | < d.l. | < d.l. | 3.43 | 1.91 | < d.l. | < d.l. |
| Cell 6* | < d.l. | < d.l. | < d.l. | 1.31 | < d.l. | 88.6 | < d.l. | < d.l. | 2.64 | < d.l. | < d.l. | 3.25 | 3.28 | < d.l. | < d.l. |
| **Receiving chamber (µg/L)** | | | | | | | | | | | | | | | |
| Cell 1 | < d.l. | < d.l. | < d.l. | < d.l. | 1.68 | < d.l. | < d.l. | < d.l. | 0.80 | < d.l. | < d.l. | 3.93 | 1.09 | < d.l. | < d.l. |
| Cell 2 | < d.l. | < d.l. | < d.l. | < d.l. | < d.l. | 29.8 | < d.l. | < d.l. | 5.08 | < d.l. | < d.l. | 3.94 | 1.74 | < d.l. | < d.l. |
| Cell 3 | 15.2 | < d.l. | < d.l. | < d.l. | 4.96 | 19.8 | < d.l. | < d.l. | 0.84 | < d.l. | < d.l. | 3.79 | 1.19 | < d.l. | < d.l. |
| Cell 4 | < d.l. | < d.l. | < d.l. | < d.l. | 2.84 | 14.1 | < d.l. | < d.l. | 0.76 | < d.l. | < d.l. | 3.70 | 1.68 | < d.l. | < d.l. |
| Cell 5* | < d.l. | < d.l. | < d.l. | < d.l. | < d.l. | 14.2 | < d.l. | < d.l. | 2.25 | < d.l. | < d.l. | 4.38 | 1.19 | < d.l. | < d.l. |
| Cell 6* | < d.l. | < d.l. | < d.l. | < d.l. | < d.l. | 14.9 | < d.l. | < d.l. | 1.22 | < d.l. | < d.l. | 4.07 | 1.20 | < d.l. | < d.l. |
| **Receiving chamber Wash (µg/L)** | | | | | | | | | | | | | | | |
| Cell 1 | 36.5 | < d.l. | < d.l. | 0.58 | < d.l. | 22.6 | < d.l. | < d.l. | 24.7 | < d.l. | < d.l. | 2.92 | 2.11 | < d.l. | < d.l. |
| Cell 2 | 38.7 | < d.l. | < d.l. | 0.63 | < d.l. | 24.4 | < d.l. | < d.l. | 2.69 | < d.l. | < d.l. | 2.93 | 2.99 | < d.l. | < d.l. |
| Cell 3 | < d.l. | < d.l. | < d.l. | < d.l. | < d.l. | < d.l. | < d.l. | < d.l. | 1.91 | < d.l. | < d.l. | 2.91 | < d.l. | < d.l. | < d.l. |
| Cell 4 | 27.7 | < d.l. | < d.l. | < d.l. | < d.l. | 26.7 | < d.l. | < d.l. | 3.89 | < d.l. | < d.l. | 4.70 | 1.68 | < d.l. | < d.l. |
| Cell 5* | < d.l. | < d.l. | < d.l. | < d.l. | < d.l. | < d.l. | < d.l. | < d.l. | 2.18 | < d.l. | < d.l. | 2.83 | 1.51 | < d.l. | < d.l. |
| Cell 6* | < d.l. | < d.l. | < d.l. | 0.99 | < d.l. | 30.8 | < d.l. | < d.l. | 0.90 | < d.l. | < d.l. | 2.80 | < d.l. | < d.l. | < d.l. |
| **Biological membrane (mg/Kg)** | | | | | | | | | | | | | | | |
| Cell 1 | 35.7 | < d.l. | < d.l. | 1.1 | < d.l. | 58.5 | < d.l. | < d.l. | < d.l. | 1.4 | < d.l. | 1.1 | < d.l. | < d.l. | 46.9 |
| Cell 2 | 50.4 | < d.l. | < d.l. | 1.4 | < d.l. | 84.0 | < d.l. | < d.l. | < d.l. | 3.2 | < d.l. | 1.0 | < d.l. | < d.l. | 41.6 |
| Cell 3 | 40.9 | < d.l. | < d.l. | 1.2 | < d.l. | 81.7 | < d.l. | < d.l. | < d.l. | 32.4 | < d.l. | 0.5 | < d.l. | < d.l. | 57.3 |
| Cell 4 | 27.7 | < d.l. | < d.l. | 1.6 | < d.l. | 57.0 | < d.l. | < d.l. | < d.l. | 16.8 | < d.l. | 0.5 | < d.l. | < d.l. | 57.3 |
| Cell 5* | 16.0 | < d.l. | < d.l. | 0.8 | < d.l. | 51.0 | < d.l. | < d.l. | < d.l. | 9.2 | < d.l. | 1.2 | < d.l. | < d.l. | 53.5 |
| Cell 6* | 10.9 | < d.l. | < d.l. | 0.4 | < d.l. | 37.4 | < d.l. | < d.l. | < d.l. | 5.0 | < d.l. | 0.8 | < d.l. | < d.l. | 57.4 |

**Table S11.** Caldas da Rainha (Assay 3, 4 and Assay 5) *–* Trace elements concentration by ICP-MS. (*Blank sample. d.l. – detection limit.)

| **Assay 3** | **Al** | **Ba** | **Co** | **Cr** | **Cu** | **Fe** | **Li** | **Mn** | **Mo** | **Ni** | **Pb** | **Rb** | **Sr** | **V** | **Zn** |
| --- | --- | --- | --- | --- | --- | --- | --- | --- | --- | --- | --- | --- | --- | --- | --- |
| **Donor chamber washing after 1 h sample contact (µg/L)** | | | | | | | | | | | | | | | |
| Cell 1 | 4,356.0 | 140.9 | 13.1 | 48.9 | 88.0 | 2934.4 | 6.02 | 409.3 | 1.3 | 8.4 | 3.73 | 5.8 | 237.6 | 18.4 | 0.0 |
| Cell 2 | 4,712.5 | 155.4 | 13.4 | 47.1 | 93.8 | 3158.3 | 5.33 | 410.5 | 1.2 | 8.6 | 3.22 | 4.1 | 252.1 | 20.2 | 15.0 |
| Cell 3 | 5,103.7 | 160.6 | 14.8 | 53.4 | 105.4 | 3,329.3 | 6.52 | 451.8 | 1.10 | 9.85 | 3.25 | 3.47 | 279.4 | 21.8 | 18.0 |
| Cell 4 | 5,588.9 | 209.5 | 16.2 | 51.36 | 124.6 | 3,537.5 | 4.70 | 503.7 | 1.13 | 10.5 | 3.53 | 3.52 | 337.3 | 25.8 | < d.l. |
| Cell 5* | < d.l. | < d.l. | < d.l. | 1.58 | 2.00 | 51.2 | < d.l. | < d.l. | 1.18 | < d.l. | < d.l. | 3.47 | 7.42 | < d.l. | < d.l. |
| Cell 6 | 5,867.6 | 229.5 | 19.5 | 55.3 | 143.0 | 3,707.4 | 5.07 | 602.0 | 0.98 | 11.4 | 4.21 | 3.37 | 349.8 | 29.6 | 11.1 |
| **Donor chamber after 24 h (µg/L)** | | | | | | | | | | | | | | | |
| Cell 1 | 27.7 | < d.l. | < d.l. | 1.77 | 2.95 | 37.62 | < d.l. | 2.32 | 1.39 | 2.28 | < d.l. | 3.77 | 8.56 | < d.l. | 111.93 |
| Cell 2 | 29.3 | < d.l. | < d.l. | 1.88 | 2.89 | 26.26 | < d.l. | 2.40 | 4.69 | 1.71 | < d.l. | 3.75 | 8.68 | < d.l. | 105.75 |
| Cell 3 | < d.l. | < d.l. | < d.l. | 1.59 | 2.53 | 14.1 | < d.l. | < d.l. | 1.34 | 1.77 | < d.l. | 3.94 | 7.41 | < d.l. | 87.3 |
| Cell 4 | 93.6 | < d.l. | < d.l. | 2.75 | 2.92 | 62.6 | < d.l. | 6.59 | 1.20 | 1.41 | < d.l. | 3.69 | 9.39 | < d.l. | 57.0 |
| Cell 5* | < d.l. | < d.l. | < d.l. | 2.20 | 2.55 | 31.7 | < d.l. | < d.l. | 1.82 | < d.l. | < d.l. | 4.04 | 14.0 | < d.l. | 70.1 |
| Cell 6 | 67.1 | < d.l. | < d.l. | 1.83 | 3.97 | 35.3 | < d.l. | 2.94 | 1.17 | 1.13 | < d.l. | 3.33 | 8.43 | < d.l. | 77.2 |
| **Receiving chamber (µg/L)** | | | | | | | | | | | | | | | |
| Cell 1 | 25.1 | < d.l. | < d.l. | 1.63 | 2.48 | 14.89 | < d.l. | < d.l. | 1.21 | < d.l. | < d.l. | 3.96 | 8.07 | < d.l. | < d.l. |
| Cell 2 | 26.2 | < d.l. | < d.l. | 1.51 | 1.93 | < d.l. | < d.l. | < d.l. | 1.15 | < d.l. | < d.l. | 3.82 | 7.33 | < d.l. | < d.l. |
| Cell 3 | 28.4 | < d.l. | < d.l. | 1.62 | 2.15 | 10.3 | < d.l. | < d.l. | 1.15 | < d.l. | < d.l. | 3.32 | 7.18 | < d.l. | < d.l. |
| Cell 4 | 25.8 | < d.l. | < d.l. | 1.64 | 1.74 | < d.l. | < d.l. | < d.l. | 1.18 | < d.l. | < d.l. | 3.27 | 7.32 | < d.l. | < d.l. |
| Cell 5* | < d.l. | < d.l. | < d.l. | 1.54 | 5.70 | 10.8 | < d.l. | < d.l. | 1.20 | < d.l. | < d.l. | 3.65 | 7.81 | < d.l. | < d.l. |
| Cell 6 | 23.1 | < d.l. | < d.l. | 1.53 | 2.09 | 12.2 | < d.l. | < d.l. | 1.35 | < d.l. | < d.l. | 3.83 | 7.30 | < d.l. | < d.l. |
| **Receiving chamber Wash (µg/L)** | | | | | | | | | | | | | | | |
| Cell 1 | < d.l. | < d.l. | < d.l. | 1.36 | 2.26 | < d.l | < d.l. | < d.l. | 1.06 | < d.l. | < d.l. | 2.95 | 7.24 | < d.l. | < d.l. |
| Cell 2 | < d.l. | < d.l. | < d.l. | 1.43 | 2.51 | < d.l. | < d.l. | < d.l. | 1.18 | < d.l. | < d.l. | 3.22 | 7.12 | < d.l. | < d.l. |
| Cell 3 | < d.l. | < d.l. | < d.l. | 1.43 | 1.96 | < d.l. | < d.l. | < d.l. | 1.18 | < d.l. | < d.l. | 2.95 | 4.97 | < d.l. | < d.l. |
| Cell 4 | < d.l. | < d.l. | < d.l. | 1.37 | 1.97 | < d.l. | < d.l. | < d.l. | 1.10 | < d.l. | < d.l. | 2.94 | 7.29 | < d.l. | < d.l. |
| Cell 5* | < d.l. | < d.l. | < d.l. | 1.45 | 2.48 | < d.l. | < d.l. | < d.l. | 1.15 | < d.l. | < d.l. | 3.15 | 7.35 | < d.l. | < d.l. |
| Cell 6 | < d.l. | < d.l. | < d.l. | 1.38 | 1.76 | < d.l. | < d.l. | < d.l. | 0.90 | < d.l. | < d.l. | 2.81 | 6.65 | < d.l. | < d.l. |
| **Biological membrane (mg/Kg)** | | | | | | | | | | | | | | | |
| Cell 1 | 18.4 | 0.6 | < d.l. | 0.6 | 1.3 | 36.2 | < d.l. | < d.l. | 0.0 | 1.3 | < d.l. | 0.0 | 0.0 | < d.l. | 39.3 |
| Cell 2 | 15.0 | 0.7 | < d.l. | 0.6 | 2.1 | 24.4 | < d.l. | < d.l. | 0.0 | 0.4 | < d.l. | 0.0 | 0.0 | < d.l. | 19.1 |
| Cell 3* | 16.1 | 1.40 | 0.059 | 1.26 | 1.32 | 20.4 | < d.l. | < d.l. | < d.l. | 1.25 | < d.l. | < d.l. | < d.l. | < d.l. | 14.1 |
| Cell 4 | 12.8 | 1.03 | 0.182 | 0.498 | 1.62 | 25.3 | < d.l. | < d.l. | < d.l. | 0.399 | < d.l. | < d.l. | < d.l. | < d.l. | 17.2 |
| Cell 5 | < d.l. | < d.l. | < d.l. | 0.6 | 0.183 | 0.844 | < d.l. | < d.l. | < d.l. | < d.l. | < d.l. | < d.l. | < d.l. | < d.l. | 0.155 |
| Cell 6 | 6.8 | 0.265 | 0.253 | 0.764 | 0.783 | 0.298 | < d.l. | < d.l. | < d.l. | 0.563 | < d.l. | < d.l. | < d.l. | < d.l. | 0.238 |

| **Assay 4** | **Al** | **Ba** | **Co** | **Cr** | **Cu** | **Fe** | **Li** | **Mn** | **Mo** | **Ni** | **Pb** | **Rb** | **Sr** | **V** | **Zn** |
| --- | --- | --- | --- | --- | --- | --- | --- | --- | --- | --- | --- | --- | --- | --- | --- |
| **Donor chamber washing after 1 h sample contact (µg/L)** | | | | | | | | | | | | | | | |
| Cell 2 | 6,272.1 | 289.7 | 20.3 | 54.2 | 172.6 | 3,653.7 | 5.55 | 698.3 | 0.63 | 14.3 | 6.21 | 3.96 | 453.0 | 35.9 | 37.8 |
| Cell 3 | 5,411.7 | 238.0 | 166 | 46.8 | 137.5 | 3,265.7 | 5.67 | 563.8 | < d.l. | 10.8 | 4.04 | 3.06 | 370.9 | 31.4 | 13.2 |
| Cell 4* | 26.2 | < d.l. | < d.l. | 1.76 | 1.23 | 23.0 | < d.l. | < d.l. | < d.l. | < d.l. | 1.72 | 3.27 | 4.50 | < d.l. | < d.l. |
| **Donor chamber after 24 h (µg/L)** | | | | | | | | | | | | | | | |
| Cell 2 | 648.8 | < d.l. | < d.l. | 11.6 | 5.96 | 600.5 | < d.l. | 16.7 | < d.l. | 2.94 | < d.l. | 2.91 | 9.35 | < d.l. | 66.6 |
| Cell 3 | 116.2 | < d.l. | < d.l. | 2.46 | 3.41 | 98.5 | < d.l. | 2.17 | 3.1 | 3.89 | < d.l. | 2.93 | 4.95 | < d.l. | 70.1 |
| Cell 4* | 23.9 | < d.l. | < d.l. | 1.71 | 2.11 | 15.0 | < d.l. | < d.l. | < d.l. | 1.55 | < d.l. | 3.20 | 4.84 | < d.l. | 71.1 |
| **Receiving chamber (µg/L)** | | | | | | | | | | | | | | | |
| Cell 2 | 31.1 | < d.l. | < d.l. | 2.24 | 2.80 | 20.8 | < d.l. | 2.24 | 0.52 | 2.50 | < d.l. | 4.11 | 5.34 | < d.l. | < d.l. |
| Cell 3 | 42.6 | < d.l. | < d.l. | 3.99 | 5.89 | 35.7 | < d.l. | 14.2 | 0.58 | 1.48 | < d.l. | 4.34 | 8.64 | < d.l. | < d.l. |
| Cell 4* | 20.2 | < d.l. | < d.l. | 2.08 | 1.91 | < d.l. | < d.l. | < d.l. | < d.l. | 1.06 | < d.l. | 4.34 | 4.85 | < d.l. | 45.1 |
| **Receiving chamber Wash (µg/L)** | | | | | | | | | | | | | | | |
| Cell 2 | 24.0 | < d.l. | < d.l. | 1.59 | 1.27 | 10.4 | < d.l. | 2.37 | 0.65 | < d.l. | < d.l. | 2.48 | 4.37 | < d.l. | 31.3 |
| Cell 3 | 30.6 | < d.l. | < d.l. | 1.75 | 1.28 | 14.5 | < d.l. | < d.l. | < d.l. | < d.l. | < d.l. | 2.47 | 4.30 | < d.l. | < d.l. |
| Cell 4* | 14.8 | < d.l. | < d.l. | 1.63 | 1.32 | < d.l. | < d.l. | < d.l. | < d.l. | < d.l. | < d.l. | 2.62 | 4.26 | < d.l. | < d.l. |
| **Biological membrane (mg/Kg)** | | | | | | | | | | | | | | | |
| Cell 2 | 23.7 | 1.539 | 0.393 | 1.49 | 2.33 | 52.9 | < d.l. | < d.l. | < d.l. | 3.84 | < d.l. | 0.531 | < d.l. | < d.l. | 25.8 |
| Cell 3 | 10.7 | 1.261 | 0.053 | 1.79 | 3.94 | 51.4 | < d.l. | < d.l. | < d.l. | 3.40 | < d.l. | 0.588 | < d.l. | < d.l. | 20.6 |
| Cell 4* | 8.7 | < d.l. | < d.l. | 0.57 | 1.36 | 34.3 | < d.l. | < d.l. | < d.l. | 2.78 | < d.l. | 0.520 | < d.l. | < d.l. | 0.485 |

| **Assay 5** | **Al** | **Ba** | **Co** | **Cr** | **Cu** | **Fe** | **Li** | **Mn** | **Mo** | **Ni** | **Pb** | **Rb** | **Sr** | **V** | **Zn** |
| --- | --- | --- | --- | --- | --- | --- | --- | --- | --- | --- | --- | --- | --- | --- | --- |
| **Donor chamber washing after 1 h sample contact (µg/L)** | | | | | | | | | | | | | | | |
| Cell 2 | 5,587.4 | < d.l. | 17.8 | 52.5 | 147.4 | 3133.1 | 5.05 | 631.6 | 1.00 | 12.1 | 4.11 | 3.0 | 421.7 | 32.1 | 17.3 |
| Cell 3 | 5,235.8 | < d.l. | 14.2 | 49.3 | 116.0 | 3,250.8 | 4.84 | 483.5 | < d.l. | 10.1 | 3.33 | 2.58 | 357.0 | 28.8 | 24.9 |
| Cell 4* | 25.0 | < d.l. | < d.l. | 1.79 | 1.02 | 23.2 | < d.l. | < d.l. | < d.l. | 1.52 | < d.l. | 2.97 | 4.19 | < d.l. | 26.4 |
| **Donor chamber after 24 h (µg/L)** | | | | | | | | | | | | | | | |
| Cell 2 | 27.0 | < d.l. | < d.l. | 1.7 | 1.8 | 19.9 | < d.l. | < d.l. | 0.0 | 3.3 | < d.l. | 3.4 | 4.6 | < d.l. | 53.9 |
| Cell 3 | 19.21 | < d.l. | < d.l. | 1.68 | 1.04 | 12.5 | < d.l. | < d.l. | 3.12 | 2.18 | < d.l. | 2.91 | 4.34 | < d.l. | 24.8 |
| Cell 4* | 18.9 | < d.l. | < d.l. | 1.84 | 4.16 | < d.l. | < d.l. | < d.l. | < d.l. | 2.37 | < d.l. | 3.30 | 4.32 | < d.l. | 33.2 |
| **Receiving chamber (µg/L)** | | | | | | | | | | | | | | | |
| Cell 2 | 20.9 | < d.l. | < d.l. | 2.8 | 2.4 | 18.1 | < d.l. | 2.59 | 0.0 | 0.0 | < d.l. | 4.8 | 5.8 | < d.l. | 0.0 |
| Cell 3 | 13.91 | < d.l. | < d.l. | 1.86 | 2.16 | < d.l. | < d.l. | < d.l. | < d.l. | 1.45 | < d.l. | 4.12 | 5.18 | < d.l. | < d.l. |
| Cell 4* | 15.7 | < d.l. | < d.l. | 1.78 | 1.79 | < d.l. | < d.l. | < d.l. | < d.l. | 1.02 | < d.l. | 5.25 | 5.40 | < d.l. | 14.5 |
| **Receiving chamber Wash (µg/L)** | | | | | | | | | | | | | | | |
| Cell 2 | 26.2 | < d.l. | < d.l. | 1.6 | 0.0 | 0.0 | < d.l. | 2.27 | 9.12 | 0.0 | < d.l. | 2.4 | 4.1 | < d.l. | 14.8 |
| Cell 3 | < d.l. | < d.l. | < d.l. | 1.52 | 1.00 | < d.l. | < d.l. | < d.l. | < d.l. | 1.15 | < d.l. | 2.52 | 3.92 | < d.l. | 11.4 |
| Cell 4* | 14.0 | < d.l. | < d.l. | 1.46 | < d.l. | < d.l. | < d.l. | < d.l. | < d.l. | < d.l. | < d.l. | 2.32 | 3.91 | < d.l. | < d.l. |
| **Biological membrane (mg/Kg)** | | | | | | | | | | | | | | | |
| Cell 2 | 2,026.9 | < d.l. | < d.l. | 1.05 | 15.8 | 29.06 | < d.l. | < d.l. | < d.l. | 1.02 | < d.l. | < d.l. | < d.l. | < d.l. | 23.40 |
| Cell 3 | 2,777.7 | < d.l. | < d.l. | 2.02 | 34.0 | < d.l. | < d.l. | < d.l. | < d.l. | 1.68 | < d.l. | < d.l. | < d.l. | < d.l. | 20.2 |
| Cell 4* | 14.00 | < d.l. | < d.l. | < d.l. | 0.95 | < d.l. | < d.l. | < d.l. | < d.l. | 1.15 | < d.l. | < d.l. | < d.l. | < d.l. | < d.l. |

**Table S12.**  Mg and Ca Mass Balance (%)

| **Cró Peloid** | **Assay 1** | **Mg** | **Ca** |
| --- | --- | --- | --- |
|  | Cell 1 | 44 | 100 |
|  | Cell 4 | 45 | 84 |
|  | Cell 5 | 51 | 63 |
|  | Cell 6 | 34 | 66 |
|  | **Assay 2** | **Mg** | **Ca** |
|  | Cell 1 | 39 | 78 |
|  | Cell 2 | 31 | 77 |
|  | Cell 4 | 37 | 58 |
| **Caldas da Rainha Peloid** | **Assay 3** | **Mg** | **Ca** |
|  | Cell 1 | 84 | 54 |
|  | Cell 2 | 36 | 27 |
|  | Cell 3 | 26 | 38 |
|  | Cell 4 | 42 | 28 |
|  | Cell 6 | 35 | 30 |
|  | **Assay 4** | **Mg** | **Ca** |
|  | Cell 2 | 25 | 33 |
|  | Cell 3 | 23 | 18 |
|  | **Assay 5** | **Mg** | **Ca** |
|  | Cell 3 | 26 | 13 |

**Table S13.** Cró and Caldas da Rainha peloid Average and Variance.

|  | | | | | | |  |
| --- | --- | --- | --- | --- | --- | --- | --- |
| *SUMMARY* | *Count* | *Sum* | *Average* | *Variance* |  |  | |
| Al | 2 | 226.04 | 113.02 | 18810.24 |  |  | |
| Ba | 2 | 1.68 | 0.84 | 0.3362 |  |  | |
| Ca | 2 | 1486 | 743 | 189728 |  |  | |
| Co | 2 | 0.24 | 0.12 | 0.0002 |  |  | |
| Cr | 2 | 3.03 | 1.515 | 3.83645 |  |  | |
| Cu | 2 | 1.04 | 0.52 | 0.0648 |  |  | |
| Fe | 2 | 174.12 | 87.06 | 13428.33 |  |  | |
| Li | 2 | 0.14 | 0.07 | 0.0072 |  |  | |
| Mg | 2 | 361 | 180.5 | 264.5 |  |  | |
| Mn | 2 | 6.24 | 3.12 | 6.6978 |  |  | |
| Mo | 2 | 0.133 | 0.0665 | 0.008065 |  |  | |
| Ni | 2 | 2.44 | 1.22 | 2.8322 |  |  | |
| Pb | 2 | 0.02 | 0.01 | 0 |  |  | |
| Sr | 2 | 1.53 | 0.765 | 0.00845 |  |  | |
| V | 2 | 0.38 | 0.19 | 0.0288 |  |  | |
| Zn | 2 | 0.48 | 0.24 | 0.045 |  |  | |
|  |  |  |  |  |  |  | |
| CRO | 16 | 1636.01 | 102.2506 | 69792.3 |  |  | |
| CR | 16 | 628.503 | 39.28144 | 12892.25 |  |  | |
|  |  |  |  |  |  |  | |
|  |  |  |  |  |  |  | |
